# A Perturbative Approach to the Solution of the Thirring Quantum Cellular Automaton

**DOI:** 10.3390/e27020198

**Published:** 2025-02-13

**Authors:** Alessandro Bisio, Paolo Perinotti, Andrea Pizzamiglio, Saverio Rota

**Affiliations:** 1 Dipartimento di Fisica, Università di Pavia, 27100 Pavia, Italy; alessandro.bisio@unipv.it (A.B.); andrea.pizzamiglio01@universitadipavia.it (A.P.); saverio.rota01@universitadipavia.it (S.R.); 2 Istituto Nazionale di Fisica Nucleare Sezione di Pavia, via Agostino Bassi, 6, 27100 Pavia, Italy

**Keywords:** quantum cellular automata, Thirring quantum cellular automaton, path sum solution, perturbative approach

## Abstract

The Thirring Quantum Cellular Automaton (QCA) describes the discrete time dynamics of local fermionic modes that evolve according to one step of the Dirac cellular automaton, followed by the most general on-site number-preserving interaction, and serves as the QCA counterpart of the Thirring model in quantum field theory. In this work, we develop perturbative techniques for the QCA path sum approach, expanding both the number of interaction vertices and the mass parameter of the Thirring QCA. By classifying paths within the regimes of very light and very heavy particles, we computed the transition amplitudes in the two- and three-particle sectors to the first few orders. Our investigation into the properties of the Thirring QCA, addressing the combinatorial complexity of the problem, yielded some useful results applicable to the many-particle sector of any on-site number-preserving interactions in one spatial dimension.

## 1. Introduction

Understanding the dynamics of interacting many-body quantum systems remains one of the paramount unsolved challenges in physics. The intricate behaviors and interdependencies within these systems render both analytical descriptions and numerical simulations exceedingly arduous. Nonetheless, the research of the last few decades has heralded an uplifting new horizon. The confluence of cutting-edge quantum technologies and the maturation of quantum information theory offers a wealth of promising investigative tools, both practical and theoretical. The rapidly increasing precision in control over quantum apparatus suggests that we will soon be able to test both novel and long-standing hypotheses, ranging from simulations in particle physics and condensed matter to exploring the very foundations of contemporary physics.

Specifically, in addressing the inefficiency of classical computers in simulating quantum systems, Quantum Cellular Automata (QCAs) delineate practical quantum algorithms for simulating the dynamics of an interacting quantum many-body system [1,2,3,4,5,6]. Quantum cellular automata are homogeneous networks of local quantum systems that abide by a translationally invariant discrete-time evolution, each interacting with a finite number of neighbors. The most interesting QCAs for quantum simulations are those that can be implemented as Finite-Depth Quantum Circuits (FDQCs) [4]. Furthermore, QCAs offer an inherent architecture for the implementation of quantum simulation hardware and for distributed quantum computation [4,7], and they are universal for quantum computation as they have been proven equivalent to both the quantum Turing machine and the circuit paradigm of quantum computation [7].

In recent years, QCAs, and Quantum Walks (QWs)—which can be regarded as the one-particle sector or first quantization counterpart of QCAs—have been investigated as instruments for simulating relativistic quantum fields and as discrete methods for studying the foundations of Quantum Field Theory (QFT) [8,9,10,11,12,13,14,15]. There are many advantages of such an approach to the foundations. From an information theoretical standpoint, quantum systems might be conceived as elementary information carriers, rather than elementary constituents of matter, circumventing the molasses of interpretational issues of quantum mechanics. With this premise, relationships between elementary quantum systems can be thought of as logical connections within an algorithm, as opposed to the usual space–time relations. Along this line, embracing the principle initially proposed by Feynman [1] and later refined by Deutsch [2], i.e., that every experiment involving a finite space–time region should be perfectly simulated by a finite quantum algorithm, we can devise physical laws as algorithms that govern the update of memory registers constituting a physical system. Within this scope, the QCA framework naturally emerges by imposing—once reframed in this context—the characteristic properties of physical laws onto algorithms, such as locality and homogeneity [16].

In recent decades, several quantum simulation schemes have been devised [17], but these are predominantly Hamiltonian-based. They rely on a discrete-space–continuous-time version of the QFT. Analog quantum systems mimicking the Hamiltonian are sought, or alternatively, staggered Trotterization (Suzuki–Trotter decomposition) is performed to obtain unitary transformations that may be implemented as a quantum circuit on a digital quantum computer, namely, a QCA implementation. Trotterized gates are close to the identity and contrast with the gates of a more general ab initio QCA model, which, conversely, not being subject to Trotterization conditions, can be highly non-trivial (possibly converging faster to physical dynamics under suitable conditions [18,19,20]). Thus, the possibility of framing algorithms not originating from conventional QFTs suggests a broader spectrum of options for simulating them via quantum computers beyond lattice QFT.

Furthermore, given that the traditional action-based approach is only surreptitiously continuous, namely, up to renormalization [21], a crucial procedure for both its mathematical amenability and physical significance, it is conceptually clearer to start from new premises based on a natively discrete theory. Indeed, QCAs exhibit manifest unitarity and possess a local formulation. Gauge invariance can be handled as outlined in [22,23,24]. Moreover, an ab initio quantum description, namely, not obtained by carrying out a quantization procedure on a classical theory, sidesteps the ambiguities that arise when striving to develop a quantum theory based on a classical action, and due to the emergent nature of the geometry of space–time, it represents an enticing arena for approaching a quantum theory of gravity [25,26,27,28].

There are, of course, also drawbacks in facing a new formulation of QFT. In the first place, being intrinsically discrete, these kinds of models were, for a long time, dismissed as incompatible with the relativistic framework that is the crucial feature of quantum fields [29]. However, in recent years, the compatibility of non-interacting QCAs with Lorentz covariance has been explicitly demonstrated in compliance with different prescriptions [26,30,31], e.g., by identifying a change of inertial frame with a symmetry of the automaton dynamics. The bounded speed of light in circuit-based quantum simulation is naturally enforced by the wiring between local quantum gates. In the second place, we should remark that we still lack a renormalization theory for QCAs [10,32,33], i.e., a procedure for identifying the correct effective (continuous) degrees of freedom and effective interactions describing the experimentally relevant large-scale behavior of a QCA (Wilson’s renormalization group framework explains why long-distance physics can be accurately described by disregarding short-distance phenomena; by absorbing small-scale effects into a few parameters of an effective field theory, the renormalization effectively decouples physics at different length scales, allowing for accurate predictions within specified limits).

The present work has two main objectives. The first one is developed in the initial sections, and consists of a review of some findings from recent years, emphasizing key concepts of fundamental significance. The second one is addressed in the following sections, which present the first application of the quantum cellular automata path sum approach to an interacting model. In detail, this paper is structured as follows. Section 2 provides a summary of the original results. In Section 3, we deliver an outline of the one-dimensional Dirac QCA [34] and of the path sum approach to its solution [35]. Section 4 provides a synopsis of the Thirring QCA [36]. In Section 5, an overarching characterization of the particle paths, grounded in the general features of Thirring QCA, is presented. Section 6 and Section 7 cover the path sum approach in the two-particle sector, with a perturbative technique in the number of interactions and in the particle mass, respectively. Finally, in Section 8, we discuss the multi-particle sector, and the three-particle case in particular.

## 2. Summary of the Results

Despite QCAs having achieved promising success in recovering free quantum field theories from the principles of information processing [11], to date, a thorough study of the interaction processes within the QCA framework is lacking, representing a largely unexplored avenue. As in QFT, this point is the main source of troubles, and the newborn theory of QCAs cannot yet count on the wealth of techniques and approaches of the standard formulation. This requires an extensive review of the theoretical tools, with the aim of either adapting them to the discrete framework or replacing them with new, more suitable techniques.

In the present study, we tackle the issue by endeavoring to expand a specific instance of the QCA approach that has proven successful in solving the non-interacting dynamics: the path sum [35]. As a case study, we consider the (1+1)-dimensional Thirring quantum cellular automaton. The interaction described by the Thirring QCA is the most general one, being both number-preserving—meaning the number of particles remains constant before and after the interaction—and on-site, requiring particles to occupy the same lattice site simultaneously to interact. Moreover, our choice is driven by three primary reasons. In the first place, the interaction-free model has been exactly treated via a path sum approach [35], providing a solid starting toolbox, and the interacting one has been analytically solved in the two-particle sector [36], allowing us to validate the results and check the accuracy of approximations. Secondarily, in the (1+1)-dimensional case, we can easily visualize the discrete evolution of the automaton on a two-dimensional space–time lattice. The evolution of a particle with specified initial and final conditions can be effectively represented by a collection of interfering paths connecting these points. Within the path sum paradigm, transition amplitudes are then obtained by summing over such paths. Finally, the Thirring model in QFT belongs to the restricted category of integrable models. Though it is not completely obvious how the Thirring QCA obtains the Thirring model in some limit, this allows us to borrow techniques from established results, and to have a source for future validation and developments.

Despite the simplicity of local rules governing QCAs, the emerging behavior exhibits increasing complexity as the evolution progresses. While this is one of the general reasons why QCAs represent a valuable tool in studying complex systems, it is also the reason why their actual classification proves to be remarkably challenging [37,38,39,40]. Within the path sum approach, and given the on-site nature of Thirring interaction, the origin of the complexity here is embodied in a comprehensive classification of all possible paths based on the number of intersections that yield an interaction. Such classification requires us to take into account both the Pauli exclusion principle and the fact that the free dynamics of the QCA univocally determines the internal state of the systems at every lattice site.

At the outset, one is led to follow a perturbative approach in the number of interactions. Having fixed the initial and final particle states, we expanded transition matrices in a sum of terms, each corresponding to a fixed number of interactions. Unfortunately, the algorithm that identifies all paths with a given number of interactions is computationally expensive. Consequently, we focused on a different perturbative approach, consisting of a power expansion in the mass parameter of the automaton. The regularity of the paths within the extremal regimes of ultra-light and ultra-heavy fermions allowed for their full enumeration at the lowest orders. We then computed the transition amplitudes in these regimes up to the third order and up to the three-particle sector.

## 3. One-Dimensional Dirac QCA

### 3.1. Derivation

The following discussion may appear to be a mere exercise, but it unveils the potential to reformulate the description of a relativistic quantum theory without initially resorting to the conventional categories of physics, such as space–time or differential equations thereof. This proves that it is feasible to develop an emergent physical semantics on top of the concept of information and its processing, without introducing theoretical physical concepts ab initio. The operational physical notions will be connected to the mathematical symbols through comparison with observations, i.e., in a similar way to how thermodynamic quantities are derived in statistical mechanics.

Indeed, the Dirac equation can be derived solely from fundamental principles of information processing, without appealing a priori to a space–time background or special relativity. It has been shown that the Dirac equation in any spatial dimension can be recovered from the large-scale dynamics of a QCA of fermionic systems satisfying linearity, unitarity, locality, homogeneity, and discrete isotropy. Here, we briefly sketch the construction (see [16] for a full derivation).

The systems are local fermionic modes labeled by the elements *g* of a countable index set *G*. Their operator algebra is generated by evaluations $\psi \left(g\right)=(\psi_{1}\left(g\right),\ldots ,\psi_{s}\left(g\right))$ of an *s*-dimensional complex vector field ψ over *G*, obeying the canonical anti-commutation relations:

$$\begin{array}{cc} \; & \left\{\psi_{a}\left(f\right),\psi_{b}\left(g\right)\right\}=\left\{\psi_{a}^{†}\left(f\right),\psi_{b}^{†}\left(g\right)\right\}=0\;,\\ \; & \left\{\psi_{a}^{†}\left(f\right),\psi_{b}\left(g\right)\right\}=\delta_{fg}\delta_{ab}\;\;\forall \;f,g\in G,\;a,b,\in \left\{1,2,\ldots ,s\right\}. \end{array}$$

By linearity, we mean that the interaction among systems is described by linear transition functions $A_{gf}$, allowing us to write the evolution of system ψ(g) by the quantum cellular automaton α (for mathematically oriented readers, ψ(g) generate a CAR C*-algebra of operators; the algebra over the entire set *G* can be strictly defined as the related quasi-local algebra, which comprises all operators that can be arbitrarily well approximated in norm topology by sequences of local operators [41]; a QCA is a *-automorphism of the quasi-local algebra that maps local operators into nearby local operators [42]; notably, this construction is guaranteed to work in the thermodynamic limit from the beginning) as

(1) $$\psi \left(g,t+1\right)=\alpha \left(\psi (g,t)\right)=\sum_{f\in S_{g}}A_{gf}\;\psi \left(f,t\right)\;,$$

with $S_{g}\subset G$ being the set of systems interacting with ψ(g). Locality is phrased by imposing $S_{g}$, being finite for every *g*. Such a locality condition introduces a causal cone notion in the emergent space–time lattice. The homogeneity requirement amounts to asking that the cardinality of $S_{g}$, as well as the set of functions ${\left\{A_{gf}\right\}}_{f\in S_{g}}$, are independent of *g*; whence we can identify $S_{g}=S$ and the functions $A_{gf}=A_{h}$ for some h∈S. Moreover, we impose $A_{gf}\neq 0$ if and only if $A_{fg}\neq 0$. We are thus assuming that the QCA evolution does not discriminate systems. It follows that the structure of the connections between systems can be regarded as the application of generators and their inverses h∈S of a discrete group, i.e., that we identify with *G*, which allows one to move from an element g∈G to another element f=hg∈G. We can then define a spatial lattice as one possible Cayley graph of this group [43]. The graph describes the interacting set of systems by taking them as the nodes of the graph, with the links corresponding to their interactions. The isotropy condition stipulates that no preferential direction exists on the lattice. Mathematically, this necessitates the presence of a permutation group Π acting on *S*, which can be faithfully and unitarily represented on the internal degrees of freedom such that $A_{\pi (h)}=U_{\pi }A_{h}U_{\pi }^{†}$, for any h∈S and π∈Π. To confine our analysis to the dynamics on an emergent flat Minkowski space–time, we assume group *G* to be virtually abelian, meaning *G* contains an abelian subgroup of a finite index. These are the groups whose Cayley graph can be quasi-isometrically embedded in Euclidean space $R^{3}$[44]. Finally, the unitarity assumption—namely, the reversibility of the algorithm—requires the local rule of a QCA to be a unitary representation of $\sum_{h\in S}A_{h}$, i.e., $\alpha \left(\psi \left(g\right)\right)=U_{S}\psi \left(g\right)U_{S}^{†}$, with $U_{S}$ being a unitary operator of the local algebra on *S*.

The quantum walk *W* associated with the QCA α acts on the Hilbert space $C^{s}⊗l^{2}\left(G\right)$ and is obtained by considering the evolution of single-particle states:

$$\begin{array}{c} \varphi_{a}\left(g\right):=\psi_{a}^{†}\left(g\right)|\Omega \rangle , \end{array}$$

where |Ω〉 denotes the vacuum state vector, which, via the GNS theorem, realizes a representation of the quasi-local algebra as concrete operator algebra acting on a separable Hilbert space [41]. The quantum walk is given by

$$\begin{array}{c} \varphi \left(g,t+1\right)=W\varphi \left(g,t\right)=\sum_{h\in S}A_{h}^{*}\;\varphi \left(h,t\right)\; \end{array}$$

and thus, it contains all the information that is needed in order to reconstruct α [16].

Any QCA of fermionic modes with G=Z, S={1,−1,0}, and s=2 —the internal degrees of freedom will be denoted with R,L—whose dynamics is linear in the field and fulfills the conditions above is unitarily equivalent to the following quantum walk on $H=C^{2}⊗l^{2}\left(Z\right)$, which is called the *Dirac quantum cellular automaton* (or Dirac Quantum Walk) in one dimension:

(2) $$\begin{array}{c} W=\left(\begin{array}{cc} nS_{R} & imI\\ imI & nS_{L} \end{array}\right)\;,\;n,m\in \left[0,1\right]\;,\;n^{2}+m^{2}=1\;,\\ \Psi \left(t+1\right)=W\Psi \left(t\right)\;,\;\Psi \left(t\right)={\left(\ldots ,\psi (x-1,t),\psi (x,t),\psi (x+1,t),\ldots \right)}^{⊺}\;,\\ \psi \left(x,t\right)=\left(\begin{array}{c} \psi_{R}\left(x,t\right)\\ \psi_{L}\left(x,t\right) \end{array}\right)\;,\;|\Psi (t)\rangle =\sum_{x\in Z}\psi \left(x,t\right)⊗|x\rangle \;, \end{array}$$

where *I*, $S_{R}=\sum_{x\in Z}|x+1\rangle \langle x|$ and $S_{L}=S_{R}^{†}$ are the identity, i.e., the right shift and the left shift operators on $l^{2}\left(Z\right)$, respectively. The field component at time t+1 and at site *x* depends only on the field components at sites *x* and x±1 at time *t* (first-neighboring scheme). Moreover, since *W* commutes with translations along the lattice, the automaton can be diagonalized in the wave-vector space, and the Dirac equation can be recovered within a suitable limit, as summarized in Appendix A.

### 3.2. Path Sum Solution

The Dirac automaton in position representation can be conveniently expressed as [35]

(3) $$W=W_{R}⊗S_{R}+W_{L}⊗S_{L}+W_{F}⊗I\;,$$

along with the following binary encoding:

$$W_{R}:=nW_{00}\;\;\;,\;\;\;W_{L}:=nW_{11}\;\;\;,\;\;\;W_{F}:=im\left(W_{01}+W_{10}\right)\;,$$

where

$$\begin{array}{c} W_{00}=\left(\begin{array}{cc} 1 & 0\\ 0 & 0 \end{array}\right)\;,\;W_{11}=\left(\begin{array}{cc} 0 & 0\\ 0 & 1 \end{array}\right)\;,\\ W_{01}=\left(\begin{array}{cc} 0 & 1\\ 0 & 0 \end{array}\right)\;,\;W_{10}=\left(\begin{array}{cc} 0 & 0\\ 1 & 0 \end{array}\right)\;. \end{array}$$

One can check that these binary matrices form a closed algebra under multiplication and satisfy a simple composition rule

(4) $$W_{ab}W_{cd}=\frac{1+{\left(-1\right)}^{b⊕c}}{2}W_{ad}=\delta_{bc}W_{ad}\;,$$

where a⊕b:=(a+b)mod2. Each step of the automaton consists of a shift $S_{K}$ acting on coordinates space according to the action of the corresponding transition matrix $W_{K}$ on the internal degrees of freedom, with $K\in \left\{R,L,F\right\}$ and $S_{F}\equiv I$. A right shift (*R*) increases the site coordinate of right modes $\psi_{R}$ by one, a left shift (*L*) decreases the site coordinate of left modes $\psi_{L}$ by the same amount, and a free shift (*F*) leaves the site coordinate unchanged upon flipping right to left mode and vice-versa. The evolution of the field over *T* time-steps is given by $W^{T}$ and can be analyzed in terms of paths connecting sites on the space–time lattice Z×N. We can then associate the generic path connecting *x* to *y* in *T* time-steps with a string $s=s_{1}s_{2}\ldots s_{T}\in {\left\{R,L,F\right\}}^{T}$ of transitions, which gives the overall transition matrix W(s):

$$W\left(s\right)W_{s_{T}}W_{s_{T-1}}\cdots W_{s_{1}}\;.$$

**Figure 1:**
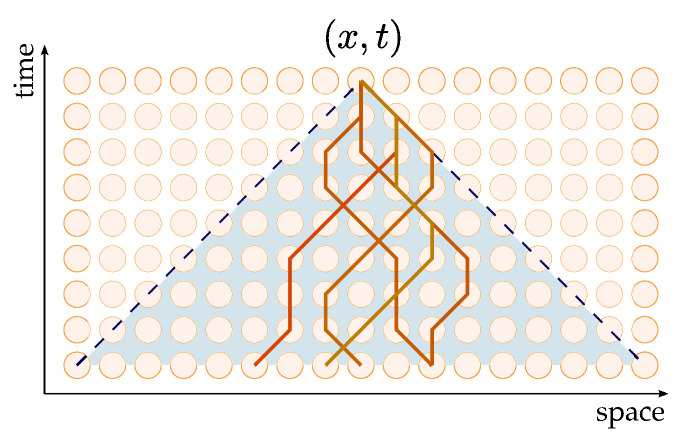
Example of paths in the past causal cone of site (x,t). The edges of the cone represent light-like paths. Space and time are expressed in arbitrary units.

The path sum approach consists of expressing the state of the system at a given time as the outcome of all the possible evolutions allowed by the dynamics, each identifying a path on the space–time lattice. This translates into expressing the state ψ(x,t) as the result of the action of W(s) on the generic initial state ψ(y,0), summed over all possible paths *s* and all points *y* in the past causal cone of site (x,t) (Figure 1):

(5) $$\psi \left(x,t\right)=\sum_{y}\sum_{s}W\left(s\right)\;\psi \left(y,0\right)\;.$$

Upon denoting with $s^{f}$ the generic path containing *f* occurrences of the *F*-transition, Equation (5) becomes

(6) $$\psi \left(x,t\right)=\sum_{y}\sum_{f=0}^{t-|x-y|}\sum_{s^{f}}W\left(s^{f}\right)\;\psi \left(y,0\right)\;.$$

In a path $s^{f}$, the *F* transitions identify f+1 slots:

$$\tau_{1}\;F\;\tau_{2}\;F\;\cdots \;F\;\tau_{f+1}$$

where $\tau_{i}$ denotes a (possibly empty) string of *R* or *L*. The composition rule in Equation (4) forbids the string *s* codifying a generic path from containing substrings of the following form:

$$\begin{array}{cccc} s_{i}s_{i-1} & =RL\;\;\;\;\; & s_{i}s_{i-1} & =LR\\ s_{i}s_{i-1}s_{i-2} & =RFR\;\;\;\;\; & s_{i}s_{i-1}s_{i-2} & =LFL \end{array}$$

as they give null transition amplitude. It follows that each sub-string $\tau_{i}$ only consists of equal letters, namely, either $\tau_{i}=RR\cdots R$ or $\tau_{i}=LL\cdots L$. Moreover, two consecutive strings $\tau_{i}$ and $\tau_{i+1}$ must contain different symbols. Therefore, all substrings $\tau_{2i}$ occupying the even slots must be of one kind, and all substrings $\tau_{2i+1}$ occupying the odd ones must be of the other kind. Exploiting the algebra (4) and relying on combinatorial arguments, we can ultimately express the state of the field at (x,t) as

(7) $$\psi \left(x,t\right)=\sum_{y}\sum_{a,b\in \left\{0,1\right\}}\sum_{f=0}^{t-|x-y|}\;\left[\alpha \left(f\right)\;c_{ab}\left(f\right)\;W_{ab}\right]\;\psi \left(y,0\right)\;,$$

with

$$\alpha \left(f\right)={\left(im\right)}^{f}\;n^{t-f}$$

and the coefficients $c_{ab}\left(f\right)$ accounting for the number of strings $s^{f}$ corresponding to paths giving $W_{ab}$ as the total transition matrix

cab(f)=μ+−νf−12−νμ−+νf−12+ν,

where

$$\nu =\frac{ab-\bar{a}\bar{b}}{2}\;\;\;\;\;\;\;\mu_{\pm }=\frac{t\pm (x-y)-1}{2}\;,$$

and $c_{aa}\left(2k+1\right)=c_{a\bar{a}}\left(2k\right)=0$ for k≠0, with $\bar{a}=a⊕1$. By definition, we set $c_{aa}\left(0\right)=1$ as the related path will be a right or a left shift at any step, corresponding to a light-like path. The analytical solution of the Dirac automaton (7) can also be expressed in terms of Jacobi polynomials by explicitly computing the sum over *f* [35].

## 4. Thirring QCA

In order to obtain an interacting quantum walker, we must consider unitary transformations that are non-linear in their degrees of freedom [24]. Non-linearity has a natural motivation from discreteness of the evolution. While in a framework where time is continuous, it is meaningful to track the evolution of the Hilbert space basis of a local system, the discrete steps of QCA evolution lack an inherent method to compare the local basis at subsequent times. Consequently, one needs to allow for a free misalignment, introducing a local unitary evolution at each time-step—preceding the linear one—that is non-linear in the fermionic modes, while preserving topological symmetries. Notice that the misalignment might follow the linear evolution rather than preceding it. In fact, both cases can be reduced to each other via a basis change of *W*, making the choice completely arbitrary and irrelevant.

We then introduce the most general on-site number-preserving interaction in the one-dimensional Dirac QCA, particularly focusing on the two-particle sector. This type of interaction characterizes a few notable integrable quantum systems [45,46,47,48], such as Hubbard’s [49] and Thirring’s [50] models. Hence, the present model was deemed the *Thirring quantum cellular automaton* [36]. When discussing non-interacting particles, the one-particle sector completely specifies the dynamics. In our context, the evolution of *N* free Dirac fermions is described by the operator $W_{N}:=W^{⊗N}$ acting on the Hilbert space $H^{⊗N}$, obtained by a tensor power of the single-particle evolution (2). The Thirring QCA is subject to the most general non-trivial on-site coupling J(χ), which preserves the number of particles. It is proved [51] that such an interaction takes the following form:

(8) $$J\left(\chi \right):=\exp\{i\;\chi \;\sum_{x}n_{R}\left(x\right)n_{L}\left(x\right)\;\},$$

where $n_{a}\left(x\right)=\psi_{a}^{†}\left(x\right)\psi_{a}\left(x\right)$ is the number operator at site *x* with internal state $a\in \left\{R,L\right\}$, and $\chi \in \left[-\pi ,\pi \right]$ is the automaton coupling constant. The single-step evolution consisting of both the free evolution and the on-site interaction, henceforth referred to as *Thirring QCA*, is therefore given by

(9) U:=WJ(χ),

where $W={⨁}_{N}W_{N}$ is the free evolution. The local non-linear coupling, represented as the exponential of *i* times a Hamiltonian, shares the same generator as the coupling found in the Hubbard and Thirring models, both of which are known to be integrable. Despite this similarity, the current cellular automaton fundamentally differs from these models due to its discrete time evolution. This discreteness leads to significant differences in the solutions of the dynamics. Already in the two-particle sector, one can see a broader spectrum of scattering states and the existence of bound states for any value of the total momentum [36,52]. Consequently, the question of the integrability of the Thirring QCA naturally arises. All known quantum integrable systems are solved via the Bethe ansatz. This method consists of solving the two-particle dynamics, formulating an ansatz for the solution of the *N*-particle case based on the two-particle solution, and verifying that the ansatz provides all the solutions. However, the periodic quasi-energy spectrum resulting from the discrete nature of time evolution in the Thirring QCA precludes the straightforward application of the conventional *N*-particle ansatz, leaving the full integrability of the Thirring QCA unsolved. The exact solution in the two particle sector was derived in Ref. [36] and is summarized in Appendix B.

## 5. Mathematical Tools

**Figure 2:**
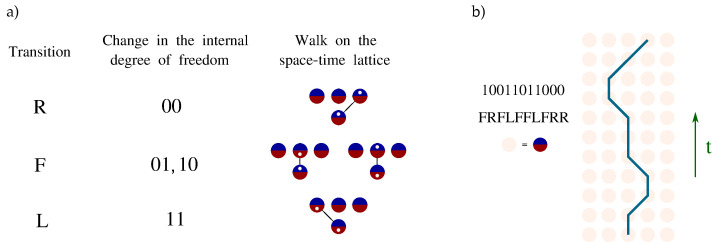
(**a**) Correspondence between different ways of representing the evolution of one particle at each step of the automaton. Each site accommodates two internal degrees of freedom: 0, or right mode (blue top half); and 1, or left mode (red bottom half). (**b**) Instance of the three representations for a path. The strings, whether representing lattice transitions or internal degrees of freedom, are ordered with time progressing from left to right, while on the lattice, time flows from bottom to top.

This section introduces mathematical tools for analyzing and classifying particle paths in the interacting scenario. Detailed proofs can be found in Appendix C. The central result we present here is that, given localized initial conditions of the particle, the free dynamics of the automaton univocally determines its state at each site of the space–time lattice. Combined with the Pauli exclusion principle, this yields necessary boundary conditions for particles to interact at any space–time point. We denote by bit 0 the right mode of the field and by bit 1 the left mode. The evolution path of a single particle can thus be described both with a string of transitions, comprising symbols R,L,F, and with a binary string associated with the internal state of the particle at each time step. Unlike the encoding in terms of R,L,F, where, e.g., substrings LR, RL, RFR, or LFL are prohibited by the algebra of the transition matrices, the main advantage of employing binary strings lies in the fact that any sequence of 0s and 1s identifies a valid path. We begin by defining a mapping between binary strings of internal degrees of freedom and the corresponding strings of translations on the lattice, which serves to elucidate the argument (Figure 2).

**Definition** **1.** *Let M be a map associating a pair of bits, encoding the state of the particle for two subsequent time steps, with the corresponding translation on the lattice*

$$\begin{array}{cc} M\;\;:\;\;{\left\{0,1\right\}}^{2} & \to \left\{R,L,F\right\}\\ b_{1}b_{2} & \mapsto M_{b_{1}b_{2}} \end{array}$$

*according to the following rules:*

$$M_{00}=R\;\;\;,\;\;\;M_{11}=L\;\;\;,\;\;\;M_{01}=M_{10}=F\;.$$

**Lemma** **1.** *There is a *1*-to-*1* correspondence between the binary strings of subsequent internal degrees of freedom $b=b_{0}\cdots b_{T}$ and the strings of translations $s=s_{1}\cdots s_{T}$, where $s_{k}=M_{b_{k-1}b_{k}}$.*

We now introduce a function that computes the spatial displacement associated with each single-step transition (R,L, or *F*) so that, given a string of translations, the resulting displacement between the starting and ending points can be evaluated as the sum of the displacements relative to each of its elements. This allows us to define the set $B_{\bar{x}}^{T}$ containing the strings of bits associated with a path connecting the states $|b_{0}^{x},\;x_{0}\rangle ,\;|b_{T}^{x},\;x_{T}\rangle \in H$ in *T* time steps.

**Definition** **2.** *Let $\bar{x}:=\left(x_{0},x_{T},b_{0}^{x},b_{T}^{x}\right)$ with $x_{0},x_{T}\in Z$ and $b_{0}^{x},b_{T}^{x}\in \left\{0,1\right\}$. The set of strings of bits representing a path connecting the state $|b_{0}^{x},\;x_{0}\rangle$ to $|b_{T}^{x},\;x_{T}\rangle$ in T time steps is given by*

$$\begin{array}{c} B_{\bar{x}}^{T}:=\left\{b^{x}=b_{0}^{x}\cdots b_{T}^{x}\in {\left\{0,1\right\}}^{T+1}\;s.t.\;\sum_{i=0}^{T-1}\Delta \left(M_{b_{i}^{x}b_{i+1}^{x}}\right)=x_{T}-x_{0}\right\}\;, \end{array}$$

*with *Δ* being the displacement operator*

(10) $$\begin{array}{cc} \Delta :\left\{R,L,F\right\} & \to \left\{0,\pm 1\right\}\\ M_{ab} & \mapsto \Delta \left(M_{ab}\right)={\left(-1\right)}^{ab}\left[1-(a⊕b)\right]\;. \end{array}$$

We show that any permutation of the internal bits of a string does not change the extreme points of the associated path.

**Lemma** **2.** *Let $b_{0}^{x}\;\tilde{b}\;b_{T}^{x}=b\in B_{\bar{x}}^{T}\;$, then $b_{0}^{x}\;\Pi \left(\tilde{b}\right)\;b_{T}^{x}=b^{\prime }\in B_{\bar{x}}^{T}$ for any permutation *Π*.*

Thus, the number of 0 s and 1 s contained in a binary string is related to the space–time coordinates of the initial and final sites of the path it describes. This relationship is made explicit in Lemma 3, which is fundamental for the derivation of all subsequent results.

**Lemma** **3.** *The number of ones contained in the string $b=b_{0}\cdots b_{T}\in {\left\{0,1\right\}}^{T+1}$ is called the weight w(b) of the string b*

$$\begin{array}{cc} w:{\left\{0,1\right\}}^{T+1} & \to N\\ b & \mapsto w\left(b\right)=\sum_{i=0}^{T}b_{i}\;. \end{array}$$

*Let $b\in B_{\bar{x}}^{T}$; then,*

(11) $$w\left(b\right)=\frac{1}{2}\left[T+\left(x_{0}-x_{T}\right)+b_{0}^{x}+b_{T}^{x}\right]\;.$$

**Figure 3:**
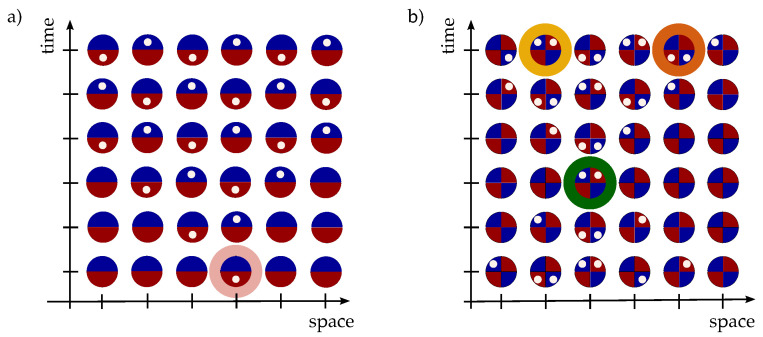
(**a**) Representation of the evolution of the internal state of a single particle at each time step upon choosing its initial state. (**b**) Representation of the evolution of the internal states of two particles.

Equation (11) highlights the interplay between the internal degrees of freedom of the particle and its space–time coordinates at any given point in space and time, given its boundary conditions. Figure 3a represents the evolution of the internal state of a single particle at each time step upon choosing its initial state (highlighted in pink).

**Lemma** **4.** *Given the state of a particle at time t, the internal degree of freedom of the particle at any site of the space–time lattice for t≠0 is univocally determined.*

This result, along with Lemma 3, implies that all binary strings connecting $|b_{0}^{x},\;x_{0}\rangle$, $|b_{T}^{x},\;x_{T}\rangle \in H$ have the same weight, namely,

$$w\left(b\right)=w\left(b^{\prime }\right)\;\;\;\;\;\forall \;b,b^{\prime }\in B_{\bar{x}}^{T}\;.$$

In turn, this implies that two such strings are connected by a permutation.

Let us now introduce a second particle. Since we are considering particles with fermionic statistics, they obey the Pauli exclusion principle. Regarding the present QCA, this translates into the requirement that no more than two particles can share the same space–time coordinate, and whenever two particles happen to be on the same site, their internal degrees of freedom must be opposite. To keep track of the relative position of the two particles along their evolution, we introduce the following definition.

**Definition** **3.** *Let (x,t) and (y,t) be the space–time coordinates of particles A and B, respectively. Their relative position at time step t is then defined as $\delta_{t}:=x-y$.*

Due to the on-site nature of the Thirring interaction (8), a necessary condition for two particles to interact at step *t* is that they occupy the same site, i.e., $\delta_{t}=0$, with opposite internal degrees of freedom. This observation, together with Equation (11), leads to a necessary condition on the initial states for an interaction to occur at a given point in space and time. For instance, consider the scenario represented in Figure 3b, where the evolution of two particles is depicted. Each site can accommodate two particles, each carrying two internal degrees of freedom. Hence, we divide each site into four quadrants: each particle disposes of a red quadrant and a blue one. Suppose the particles interact at a given site (highlighted in green). By applying the usual rules for the free evolution (which is unitary and thus reversible), we can fill the containers across the lattice with the internal state of the both particles. Notice that at the initial time step (corresponding to the bottom row), whenever the second particle is in the same (opposite) internal state as the first one, the two are separated by an odd (even) number of sites. The present graphical representation of the particle Hilbert space offers a valuable tool, applicable to many-particle systems and potentially accommodating dynamics that do not conserve particle number, with each site hosting a Fock space.

**Lemma** **5.** *Consider two particle paths whose starting points at time t=0 are separated by $\delta_{0}$ lattice sites. The particles can interact at some time t≠0 iff their internal degrees of freedom at t=0 are*

- *Opposite if $\delta_{0}$ is even;*
- *Equal if $\delta_{0}$ is odd.*

*We denote by $H_{\mathrm{int}}\subset H^{⊗2}$ the set of states satisfying such condition. Given a two-particle state $|b,\;x\rangle |b^{\prime },\;y\rangle \in H_{\mathrm{int}}$, $U_{2}|b,\;x\rangle |b^{\prime },\;y\rangle \in H_{\mathrm{int}}$.*

**Corollary** **1.** *Given two strings $b^{x}\in B_{\bar{x}}^{T}$ and $b^{y}\in B_{\bar{y}}^{T}$ with $|b_{0}^{x},\;x_{0}\rangle |b_{0}^{y},\;y_{0}\rangle \in H_{\mathrm{int}}$, there is no string connecting $|b_{0}^{x},\;x_{0}\rangle$ to $|b_{T}^{y},\;y_{T}\rangle$ nor $|b_{0}^{y},\;y_{0}\rangle$ to $|b_{T}^{x},\;x_{T}\rangle$.*

For an instance of Corollary 1, consider Figure 3b. The particles in the yellow and orange sites belong to the interacting subspace $H_{int}$. If we measure the internal state at the yellow (orange) site and find it in the blue (red) quadrant, we know it is the state of the particle evolving in the left (right) quadrants, and vice-versa if we measure it in the red quadrant. Thus, there is no ambiguity in discerning the two particles based on their internal state.

To conclude this section, one last result is showcased, which will be useful in classifying the paths of interacting particles based on their relative position at the beginning and the end of a process.

**Lemma** **6.** *If an interaction occurs at step t, then*

(12) $$\delta_{t-1}\delta_{t+1}\leq 0\;.$$

Upon fixing the initial and final states in $H_{int}$ for an interaction process, Lemma 6 allows us to establish if an even or odd number of interactions occurred.

**Corollary** **2.** *Let $\delta_{0}$ and $\delta_{T}$ be the relative position of particles at time 0 and T, respectively, for a T-step evolution. Then,*

- *If $\delta_{0}\delta_{T}>0$, the particles interact an even number 2k of times with $k\in N_{0}$ (they may not interact at all);*
- *If $\delta_{0}\delta_{T}<0$, the particles interact an odd number 2k+1 of times with $k\in N_{0}$ (they must interact at least once).*

The case $\delta_{0}\delta_{T}=0$ will be discussed in Section 7.

## 6. Perturbative Approach in the Number of Interactions

We now turn to the description of the perturbative techniques developed for the evaluation of transition matrices in the two-particle sector of the Thirring QCA. The path sum approach requires a thorough classification of all the allowed pairs of paths based on the number of intersections between them to determine how many times the on-site coupling J(χ) must be accounted for. The computational complexity of this task is exponential with respect to the size of the portion of the space–time lattice involved in the process. To reduce complexity, we adopted the following expedient, allowing for the separation of the intersections between paths and interactions of the particles, which would otherwise coincide. We write the QCA for *T* evolution steps of two particles by expressing each interaction term as J=I+(J−I), where *I* denotes the identity on the Hilbert space $H^{⊗2}$

$$\begin{array}{cc} U_{2}^{T} & =JW_{2}JW_{2}\cdots JW_{2}=\left[I+\left(J-I\right)\right]W_{2}\left[I+\left(J-I\right)\right]W_{2}\cdots \left[I+\left(J-I\right)\right]W_{2}\;. \end{array}$$

**Figure 4:**
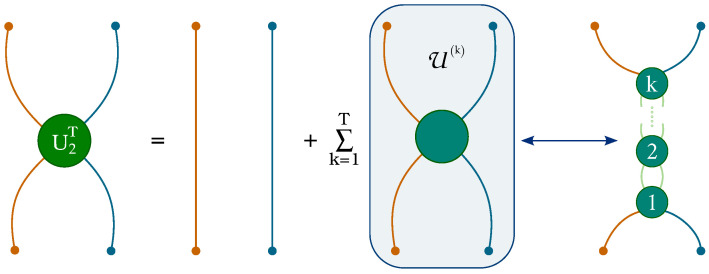
We separate the total *T*-step process in a free evolution, given by the matrices $W_{2}$ (lines), and an interacting one, described by the terms $U^{\left(k\right)}$. Each $U^{\left(k\right)}$ contains *k* interactions (circles).

We then group terms based on the number of interactions within them (Figure 4), thus obtaining

(13) $$U_{2}^{T}=W_{2}^{T}+\sum_{k=1}^{T}U^{\left(k\right)}\;,$$

where $W_{2}^{T}$ corresponds to the free evolution, while $U^{\left(k\right)}$ denotes the contribution of paths containing *k* interaction operators (J−I):

$$U^{\left(k\right)}:=\sum_{\bar{\alpha }\in A_{k}}W_{2}^{\alpha_{0}}\left(J-I\right)W_{2}^{\alpha_{1}}\left(J-I\right)\cdots W_{2}^{\alpha_{k-1}}\left(J-I\right)W_{2}^{\alpha_{k}}$$

with

$$A_{k}:=\left\{\bar{\alpha }=\left(\alpha_{0},\ldots ,\alpha_{k}\right)\in N^{k+1}\;\;\;s.t.\;\;\sum_{i=0}^{k}\alpha_{i}=T\;\;\;\wedge \;\;\alpha_{i}\neq 0\;\;\forall \;i>0\right\}\;.$$

We remark that within this context, the term *perturbative* has a slightly different meaning than the usual one. We are indeed power expanding in the number of interaction terms, but we are not claiming that some contributions are negligible compared to others. The result of the calculation is exact. We point out that terms of higher powers can significantly correct terms of lower powers in the expansion Equation (13). One recovers the usual perturbative approach when $|e^{i\chi }-1|\ll 1$; thus the sum can be regarded as a perturbative expansion in the coupling χ and can be truncated to a given order, leading to progressively more accurate approximations.

**Figure 5:**
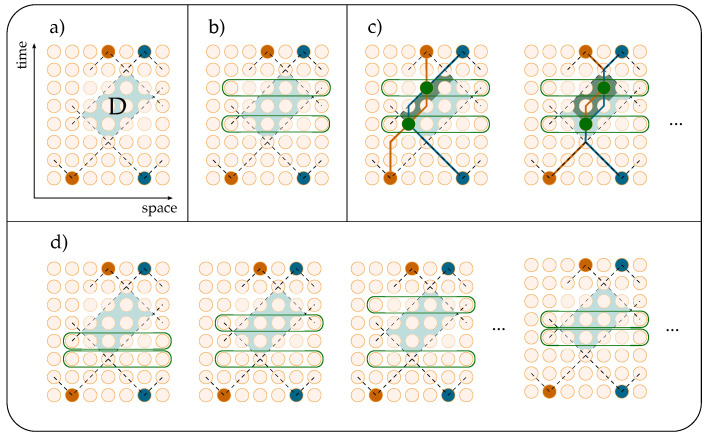
Algorithm for the computation of $U^{\left(k\right)}$ for the case k=2 at fixed initial and final particle states: (**a**) The region *D* where interactions can occur; (**b**) example of choice of two time steps wherein the interactions occur; (**c**) sum of all possible causally connected interaction sites (green dots and darker background) for the given choice of time steps; (**d**) sum of all possible ways of choosing time steps within region *D*.

To evaluate the contribution of $U^{\left(k\right)}$ to the total transition matrix, we devised the following algorithm (Figure 5). Given the initial and final particle states (orange and blue dots in Figure 5), undertake the following steps: (a) find the region *D* where interactions can occur; (b) select *k* time steps wherein the interactions occur; (c) fix the interaction sites (green dots) at each chosen time step, then evaluate the transition matrix associated with paths interacting at these sites and sum over all ways of choosing causally connected (darker background) interaction sites; (d) sum over all possible ways of choosing time steps within region *D*. The sums in items (c) and (d) run over the set of solutions of a linear system of equations obtained by redistributing the weight of the binary strings associated with the particles’ evolution into the respective k+1 sub-strings connecting the interaction vertices. This is made possible by Lemma 2, as long as the first and last bits of the strings are kept fixed. Let us stress that $U^{\left(k\right)}$ describes the evolution of paths which, in general, intersect a number of times greater than *k*. Fixing *k* time steps of interaction, the particles evolve freely between these interaction sites. That is, intersections that occur at times different from those specified do not yield interactions.

As we are considering fermionic fields, physical states belong to the anti-symmetric subspace $H_{A}^{⊗2}$ of the two particles’ Hilbert space $H^{⊗2}$. We denote by $U_{A}^{\left(k\right)}$ the projection of $U^{\left(k\right)}$ onto $H_{A}^{⊗2}$. Since the evolution $U_{2}$ commutes with the projector onto the anti-symmetric subspace, one can equivalently project after *T* steps of evolution or at each time step. In our context, it is convenient to choose the latter option; hence, we will focus on $U_{A}^{\left(k\right)}$ instead of $U^{\left(k\right)}$ in the following

$$\begin{array}{cc} U_{A}^{\left(k\right)}= & \frac{1}{4}\left(I-E\right)\;U^{\left(k\right)}\left(I-E\right)\\ = & \frac{1}{2^{k}}\;\sum_{\bar{\alpha }\in A_{k}}\left(I-E\right)W_{2}^{\alpha_{0}}\left(I-E\right)\left(J-I\right)\left(I-E\right)W_{2}^{\alpha_{1}}\left(I-E\right)\left(J-I\right)\left(I-E\right)\cdots \\ \; & \;\;\;\;\;\;\;\cdots W_{2}^{\alpha_{k-1}}\left(I-E\right)\left(J-I\right)\left(I-E\right)W_{2}^{\alpha_{k}}\left(I-E\right)\\ = & {\left(e^{i\chi }-1\right)}^{k}\;\frac{\left(I-E\right)}{2^{k}}\;\sum_{\bar{\alpha }\in A_{k}}\left[\prod_{n=0}^{k}{\left(W_{i_{n}j_{n}}⊗W_{l_{n}m_{n}}\right)}^{\alpha_{n}}\left(I-E\right)\right]\;, \end{array}$$

with (I−E)/2 the projector onto $H_{A}^{⊗2}$; $E=\frac{1}{2}\left[\sum_{i=0}^{3}\sigma_{i}⊗\sigma_{i}\right]⊗P⊗\int_{B}dp|p\rangle \langle p|\;$ the exchange operator in the hybrid basis (defined in Appendix B); $\sigma_{0}=I_{2}$ and $\sigma_{i}$ i=1,2,3 the Pauli matrices; and P|y〉=|−y〉 the parity operator on relative position.

Let $|b_{0}^{x},\;x_{0}\rangle |b_{0}^{y},\;y_{0}\rangle ,\;|b_{T}^{x},\;x_{T}\rangle |b_{T}^{y},\;y_{T}\rangle \;\in H_{int}$ be the initial and final states of the two particles for a *T*-step evolution. By some tedious albeit straightforward calculations, $U_{A}^{\left(k\right)}$ can be expressed more compactly by exploiting the algebra of the binary matrices $W_{ij}$ and the fact that $E^{2}=I$, resulting in a product of Kronecker δs linking the internal degrees of freedom at each evolution step. Enforcing the δs on the sums over paths contributing to $\langle b_{T}^{x},\;x_{T}|\langle b_{T}^{y},\;y_{T}|U_{A}^{\left(k\right)}|b_{0}^{x},\;x_{0}\rangle |b_{0}^{y},\;y_{0}\rangle$, one can show that $U_{A}^{\left(k\right)}$ is proportional to $2^{k-1}\left(I-E\right)\left(W_{b_{0}^{x}b_{T}^{x}}⊗W_{b_{0}^{y}b_{T}^{y}}\right)\left(I-E\right)$ with a coefficient accounting for the coupling and the multiplicity of such paths. The mentioned sums are those of free theory (Equation (7)). In the remaining section, we provide an exact schematic formula for $U_{A}^{\left(k\right)}$. Let $U_{A,\;V}^{\left(k\right)}$ denote the contribution to $U_{A}^{\left(k\right)}$ coming from paths with fixed interaction vertices ${\left\{v_{i},\tau_{i}\right\}}_{i=1}^{k}$, where $V={\left(\left(v_{1},\tau_{1}\right),\ldots ,\left(v_{k},\tau_{k}\right)\right)}^{⊺}$. Namely, $U_{A,\;V}^{\left(k\right)}$ is the output of item (c) of the algorithm outlined in Figure 5 (the algorithm can be applied the same way to compute either $U^{\left(k\right)}$ or $U_{A}^{\left(k\right)}$). We define

$$\begin{array}{cc} M(x,y,t,v,t^{\prime }) & :=\;\sum_{f_{x}=0}^{|t^{\prime }-t\left|-|v-x\right|}\;\;\sum_{f_{y}=0}^{|t^{\prime }-t\left|-|v-y\right|}\alpha \left(f_{x}\right)\;\alpha \left(f_{y}\right)\;c_{\phi \left(x,t\right)\phi \left(v,t^{\prime }\right)}\left(f_{x}\right)\;c_{\phi \left(y,t\right)\bar{\phi (v,t^{\prime })}}\left(f_{y}\right)\;,\\ N(v,t,v^{\prime },t^{\prime }) & :=\;\sum_{f,f^{\prime }=0}^{|t^{\prime }-t\left|-\right|v^{\prime }-v|}\alpha \left(f\right)\;\alpha \left(f^{\prime }\right)\;c_{\phi \left(v,t\right)\phi \left(v^{\prime },t^{\prime }\right)}\left(f\right)\;c_{\;\bar{\phi (v,t)}\;\bar{\phi (v^{\prime },t^{\prime })}}\left(f^{\prime }\right)\;,\\ \phi (z,t) & :=\;b_{0}^{x}⊕\left(z-x_{0}+t\right)\;, \end{array}$$

then, $M(x_{0},y_{0},0,v_{1},t_{1})$ ($M(x_{T},y_{T},T,v_{k},t_{k})$) is the multiplicity of paths connecting the initial (final) states to the first (last) interaction vertex, while $N(v_{i},t_{i},v_{i+1},t_{i+1})$ is the multiplicity of paths going from one interaction vertex to the following one. Therefore,

$$\begin{array}{cc} U_{A,\;V}^{\left(k\right)}= & \;{\left(e^{i\chi }-1\right)}^{k}\;\left[M\left(x_{0},y_{0},0,v_{1},t_{1}\right)\prod_{i=1}^{k-1}N\left(v_{i},t_{i},v_{i+1},t_{i+1}\right)\;M\left(x_{T},y_{T},T,v_{k},t_{k}\right)\right]\\ \; & \times \;\frac{1}{2}\left(I-E\right)W_{b_{0}^{x}b_{T}^{x}}⊗W_{b_{0}^{y}b_{T}^{y}}\left(I-E\right)\;, \end{array}$$

where for k=1, the *N* product reads 1. Finally, for items 4 and 5 of the algorithm in Figure 5, we can write

(14) $$U_{A}^{\left(k\right)}=\sum_{V\in S}U_{A,\;V}^{\left(k\right)}\;,$$

with S being the set of all choices of *k* causally connected interaction vertices.

In conclusion, we tried to devise a set of rules to readily compute the transition amplitudes for interaction processes similar to what is undertaken in QFT with the Feynman rules. Our procedure allows for the classification of all interacting diagrams containing a fixed number of interactions, corresponding to the chosen perturbative order when $|e^{i\chi }-1|\ll 1$. However, our approach needs to face an additional notable challenge: unlike the canonical formulation of the path integral in QFT, relying on the principle of stationary action, which prescribes that all paths (even non-physical ones) are taken into account when evaluating transition amplitudes, our Hamiltonian-free approach does not enjoy this possibility. Restricting to physical paths requires the identifying of all permitted space–time coordinates for each interaction site so that they are causally connected. This translates into a search problem involving the iterative determination of the set S of Equation (14). Unfortunately, the number of searches required grows exponentially with the size of the space–time region of interest, resulting in a computationally expensive algorithm. Consequently, our algorithm offers no substantial computational advantage over classical simulations of the automaton for predicting physical outcomes.

## 7. Perturbative Approach in the Mass Parameter

**Figure 6:**
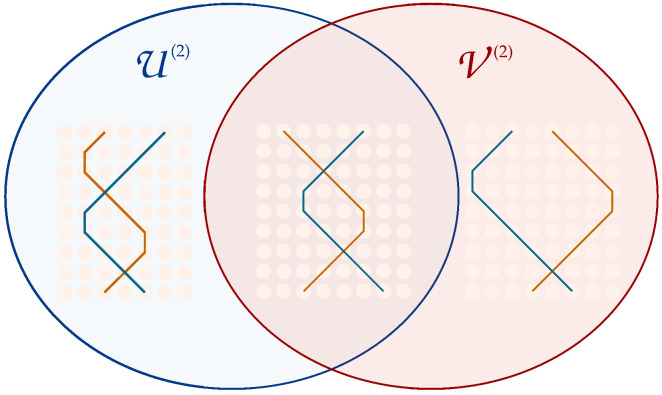
Interacting diagrams contributing to the second order in vertices expansion are highlighted in blue, while interacting diagrams contributing to the second order in mass expansion are highlighted in red.

Owing to the significant challenges encountered in developing a perturbative approach in the number of interactions, we focused on physical regimes characterised by reduced dynamical complexity. Within the path sum framework, this amounts to restrict to walks of high regularity, where particles tend to either consistently shift or remain stationary. Consequently, the number of paths leading to an interaction diminishes, enabling their manual enumeration. Figure 6 illustrates the distinction between classifying diagrams in the perturbative approach based on the number of interactions versus the mass parameter. We can identify two simplified dynamical regimes depending on the magnitude of the mass parameter *m* of the Dirac QCA (Equation (Section 3)):

- If m≪1, the off-diagonal terms in *W* contribute minimally to the dynamics, resulting in paths where the particle shifts at almost every step;
- If m≈1, the contribution of the off-diagonal terms dominates the dynamics, resulting in paths where the particle rarely shifts.

In order to exploit this feature, it is convenient to think of the evolution in terms of the string of transitions: light particles are associated with strings containing very few *F*-type transitions, while heavy particles are associated with strings that mainly consist of *F*-type transitions. Therefore, the perturbative approach in the mass parameter translates into a perturbative approach in the number *f* of *F*-type transitions.

We were able to compute the transition amplitudes for two(three)-particle processes at the first few perturbative orders, both in low- and high-mass regimes. Unfortunately, the task of classifying all admissible paths becomes unfeasible for higher perturbative orders due to the rapidly increasing number of diagrams.

Generally, the transition amplitude for a *T*-step evolution can be written as the sum of two components, representing the contribution of non-intersecting and intersecting paths. For a two-particle process, we have

$$\begin{array}{cc} \langle b_{T}^{x},\;x_{T}|\langle b_{T}^{y},\;y_{T}|\; & U_{2}^{T}\;|b_{0}^{x},\;x_{0}\rangle |b_{0}^{y},\;y_{0}\rangle \\ \; & =\langle b_{T}^{x},\;x_{T}|\langle b_{T}^{y},\;y_{T}|\;G\left(T,\bar{x},\bar{y},\chi \right)\;W_{b_{0}^{x}b_{T}^{x}}⊗W_{b_{0}^{y}b_{T}^{y}}\;|b_{0}^{x},\;x_{0}\rangle |b_{0}^{y},\;y_{0}\rangle \\ \; & =G(T,\bar{x},\bar{y},\chi )\;, \end{array}$$

where the function (propagator) *G*

(15) $$G\left(T,\bar{x},\bar{y},\chi \right)=G_{0}\left(T,\bar{x},\bar{y}\right)+G_{int}\left(T,\bar{x},\bar{y},\chi \right)$$

accounts for the multiplicity and coupling strength of free $\left(G_{0}\right)$ and interacting $\left(G_{int}\right)$ paths compatible with the chosen boundary conditions $\bar{x}=\left(x_{0},x_{T},b_{0}^{x},b_{T}^{x}\right)$ and $\bar{y}=\left(y_{0},y_{T},b_{0}^{y},b_{T}^{y}\right)$. Focusing on the interacting subspace $H_{\mathrm{int}}$, we can exploit Corollary 2. When the relative position of the two particles changes sign, i.e., when $\delta_{0}\delta_{T}<0$, they must interact an odd number of times, meaning the particles interact at least once and there is no contribution from the purely free dynamics to the total transition amplitude. Conversely, when the relative position of the two particles does not change sign, i.e., when $\delta_{0}\delta_{T}>0$, they must interact an even number of times. Hence, the particles can reach their final states without interacting. In such a scenario, the total transition amplitude receives a contribution from both free and interacting dynamics. Lastly, when $\delta_{0}\delta_{T}=0$, we have $G=G_{int}$, whose contributing diagrams have a fixed parity of interactions depending on the internal degrees of freedom of the boundary states. Moreover, when $\delta_{0}=0$, we add a phase $e^{i\chi }$ to the total transition amplitude to account for the interaction within the initial particles state. Notice that adding a phase is arbitrary for the choice of the initial state and, because of the order of operators in our convention $U_{2}^{T}={\left(J\;W_{2}\right)}^{T}$, it is needed for the amplitude of processes with $\delta_{0}=0$ that $\delta_{T}=z$ be invariant under time reversal ($\delta_{0}=z,\delta_{T}=0$). In the following, we will focus exclusively on processes where the future causal cone of the initial sites intersects with the past causal cone of the final ones.

As a consequence of Lemma 4, the parity of *f* must be the same for all paths sharing the same boundary conditions. An *F*-type transition corresponds to either 01 or 10 in the binary representation. Thus, *f* can be interpreted as the number of changes in the internal degree of freedom of the particle along its path. Therefore, if a particle departs from site $x_{0}$ with an internal state $b_{0}^{x}$ and arrives at site $x_{T}$ with state $b_{T}^{x}=b_{0}^{x}$ ($\bar{b_{0}^{x}}$), an even (odd) number of changes must have occurred, meaning *f* must be even (odd). We recall that the maximum value of *f* for a path connecting $x_{0}$ to $x_{T}$ over *T* discrete-time steps is given by (Ref. [34]):

(16) $$f_{max}\left(T,\bar{x}\right)=T-\left|x_{0}-x_{T}\right|\;.$$

Then, the set of admissible values *f* for a path with given boundary conditions is given by

$$F\left(T,\bar{x}\right):=\left\{f\in N-\left\{0\right\}\;\;s.t.\;\;f=f_{max}-2n\;\;n\in N\right\}\;,$$

assuming f=0 whenever $f_{max}=0$, corresponding to a light-like path.

We are interested in classifying diagrams describing the evolution at a given perturbative order of two particles, which we assume to have the same mass. Henceforth, *f* will denote the total number of *F*-type transitions within the diagrams, i.e., the sum of those of both particles’ paths. The classes of diagrams describing a *T*-step process with boundary conditions $\bar{x},\;\bar{y}$, and f=n are defined as elements of the set

$$\begin{array}{c} F_{T,\bar{x},\bar{y}}^{\left(n\right)}:=\left\{\left(f_{1},f_{2}\right)\in F\left(T,\bar{x}\right)\times F\left(T,\bar{y}\right)\;\;s.t.\;\;f_{1}+f_{2}=n\right\}\;, \end{array}$$

where $0\leq n\leq f_{max}\left(T,\bar{x}\right)+f_{max}\left(T,\bar{y}\right)$ provides the perturbative order. This allows us to write

(17) $$\begin{array}{cc} G & =\sum_{n}G^{\left(n\right)}\;,\;G^{\left(n\right)}=G_{0}^{\left(n\right)}+G_{int}^{\left(n\right)}\;,\\ G_{*}^{\left(n\right)} & :=\;\sum_{j\in I_{n}}\;\sum_{\left(f_{1},f_{2}\right)\in F_{T,\bar{x},\bar{y}}^{\left(j\right)}}G_{*}^{\left(f_{1},f_{2}\right)}\;\mathrm{with}\;\;\;*=0,int\;,\\ I_{n} & =\left\{\begin{array}{cc} {}[0,n]\;\;\;\;\; & \mathrm{if}\;m\ll 1\\ 2T-n,2T & \mathrm{if}\;m\approx 1\;, \end{array}\right. \end{array}$$

where $G_{*}^{\left(f_{1},f_{2}\right)}$ is the propagator *G* restricted to diagrams such that the paths associated with one particle (the other) contain $f_{1}$ ($f_{2}$) *F*-type transitions (note that $G_{*}^{\left(f_{1},f_{2}\right)}=G_{*}^{\left(f_{2},f_{1}\right)}$). We observe that $G_{int}^{\left(f_{1},f_{2}\right)}$ provides information on the number C(k) of paths yielding *k* interactions for any allowed value *k*; namely, we can write

$$G_{int}^{\left(f_{1},f_{2}\right)}=\alpha \left(f_{1}\right)\;\alpha \left(f_{2}\right)\;\sum_{k}e^{ik\chi }\;C\left(k\right)\;,$$

where we recall that $\alpha \left(f\right)={\left(im\right)}^{f}\;n^{T-f}$. Taking χ=0 yields the total number of intersecting diagrams. Therefore, $G_{0}^{\left(f_{1},f_{2}\right)}$ can be obtained by the difference between the number of all paths contributing to the process (Equation (7)) and the number of interacting ones

(18) $$G_{0}^{\left(f_{1},f_{2}\right)}=\alpha \left(f_{1}\right)\;\alpha \left(f_{2}\right)\;c_{b_{0}^{x}b_{T}^{x}}\left(f_{1}\right)c_{b_{0}^{y}b_{T}^{y}}\left(f_{2}\right)-G_{int}^{\left(f_{1},f_{2}\right)}\left(\chi =0\right)\;.$$

In the next subsection we show how to compute $G_{int}^{\left(f_{1},f_{2}\right)}$ in both low-mass and high-mass regimes.

### 7.1. Classification of Interacting Diagrams

**Figure 7:**
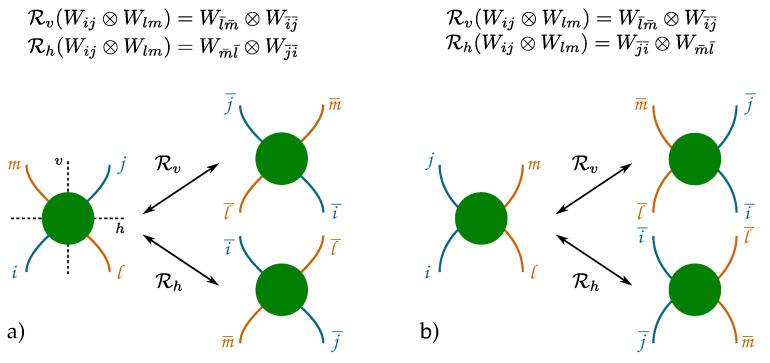
$R_{v}$ and $R_{h}$ represent on binary matrices $W_{ij}$ (lines) the reflections about the vertical *v* and horizontal *h* axes of the plane. Big circles represent the full process. Panel (**a**,**b**) shows the case where the relative position of the particles does (does not) change sign.

In this subsection we only consider diagrams involving at least one interaction, namely, those contributing to $G_{int}^{\left(f_{1},f_{2}\right)}$. Diagrams that can be obtained from each other through reflections about the vertical and horizontal axis of the plane (Figure 7) share the same multiplicity and thus give rise to the same transition amplitude.

#### 7.1.1. Low-Mass Limit

We begin by discussing the case of light particles, whose paths contain very few *F*-type transitions. In the following, we consider the classes of diagrams providing full perturbative orders n=0,1,2,3.

Let us start with the trivial case f=0. To change the direction of its path, a particle must undergo an *F*-type transition. Therefore, diagrams with $f=f_{1}+f_{2}=0$ correspond to a scenario where both particles evolve along light-like paths and intersect at one site. The resulting transition amplitude is

$$G_{int}^{\left(0,0\right)}=e^{i\chi }n^{2T}.$$

Whenever one of the two paths in the diagram is light-like, there can be at most one interaction. Indeed, the only way for the particles to interact more than once would be to shift in the same direction immediately after the first interaction. However, there is no way for this to happen while obeying both the Pauli exclusion principle and the automaton’s dynamics. Thus, for any value f≥1,

$$G_{int}^{\left(0,f\right)}=e^{i\chi }\;{\left(im\right)}^{f}\;n^{2T-f}\;c_{a\;a⊕f}\left(f\right)\;\;\;\;\;a\in \left\{0,1\right\}\;.$$

For classes (1,f−1) with f≥2, diagrams contain, at most, two interactions, one before the single *F*-type transition and one afterwards. When the relative position changes the particles must interact once, then, for these processes,

$$G_{int}^{\left(1,f-1\right)}=e^{i\chi }\;{\left(im\right)}^{f}\;n^{2T-f}\;c_{a\;\bar{a}⊕f}\left(f-1\right)\;.$$

**Figure 8:**
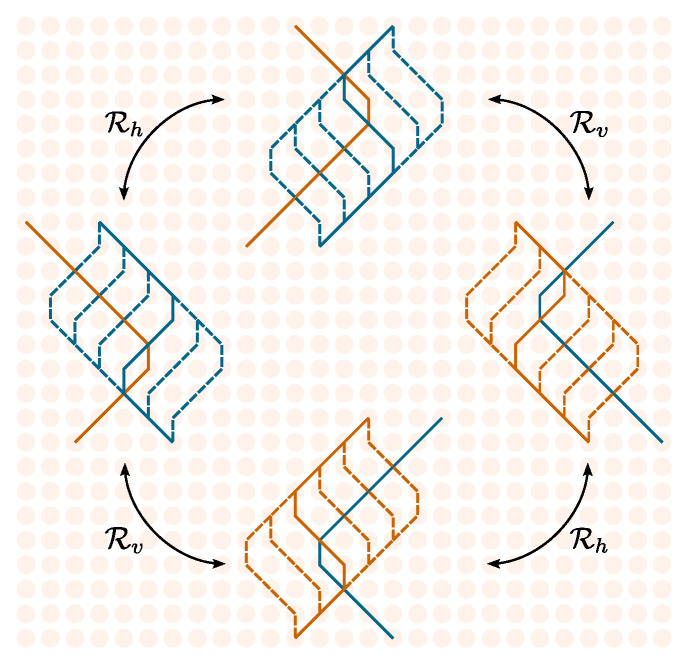
Diagrams belonging to the class (1,2) for some *T*-step process with f=3. Dashed lines represent alternative paths connecting the same extremal points; notice that not all of them yield interactions. Equation (19) accounts for this occurrence by excluding such paths from contributing to the interaction amplitude.

Conversely, when the relative position does not change, we are not able to provide a closed formula for the interacting transition amplitude. We explicitly computed the case (1,2) (Figure 8) (the case (1,1) is trivial since $c_{ab}\left(1\right)=1\;\forall a,b\in \left\{0,1\right\}$)

(19) $$G_{int}^{\left(1,2\right)}=-ie^{2i\chi }\;m^{3}\;n^{2T-3}\left\{\begin{array}{cc} \; & c_{11}\left(y_{0},y_{T},2\right)-\frac{|x_{0}-y_{0}|}{2}\;,\\ \; & c_{00}\left(y_{0},y_{T},2\right)-\frac{|x_{T}-y_{T}|}{2}\;,\\ \; & c_{11}\left(x_{0},x_{T},2\right)-\frac{|x_{T}-y_{T}|}{2}\;,\\ \; & c_{00}\left(x_{0},x_{T},2\right)-\frac{|x_{0}-y_{0}|}{2}\;. \end{array}\right.$$

**Figure 9:**
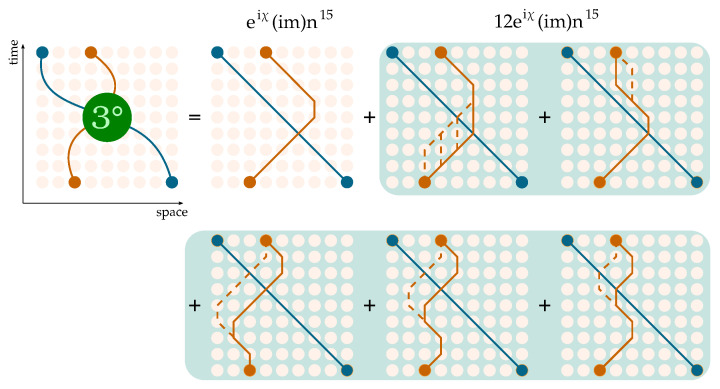
All diagrams contributing to the low-mass regime process depicted in Figure up to the third perturbative order n=3. The first diagram belongs to the class $F^{\left(1\right)}$, while highlighted in turquoise are the 12 diagrams belonging to the class $F^{\left(3\right)}$ (dashed lines represent alternative paths connecting the same extremal points).

We end this subsection with a simple example showing the explicit computation of a transition amplitude up to the third perturbative order. Consider the scenario in Figure 9 with boundary conditions $\bar{x}=\left(0,1,1,0\right),\;\bar{y}=\left(6,-2,1,1\right)$, where we set the origin in $x_{0}$ and T=8. One then easily computes the classes of contributing diagrams $F^{\left(0\right)}=\left\{\emptyset \right\},F^{\left(1\right)}=\left\{(0,1)\right\},F^{\left(2\right)}=\left\{\emptyset \right\},F^{\left(3\right)}=\left\{(0,3)\right\}$. Since we are in the scenario $\delta_{0}\delta_{T}<0$, we have

$$G≃\sum_{n=0}^{3}G^{\left(n\right)}=G_{int}^{\left(1\right)}+G_{int}^{\left(3\right)}=G_{int}^{\left(0,1\right)}+G_{int}^{\left(0,3\right)}\;.$$

From the above classification, we then obtain

$$G\left(T,\bar{x},\bar{y},\chi \right)≃e^{i\chi }\;\left(im\right)\;n^{13}\;\left[n^{2}-12m^{2}\right]\;.$$

As a cross-check, via Equations (18) and (17), one can verify that $G_{0}^{\left(3\right)}=G_{0}^{\left(0,1\right)}+G_{0}^{\left(0,3\right)}=0$.

This concludes our analysis of the low-mass regime. Up to perturbative order n=3, all diagrams included at least one path with multiplicity 1 (either a light-like path or a single *F*-type transition), simplifying the combinatorial analysis. However, this no longer holds at higher orders (f≥4 with $f_{1},f_{2}\geq 2$), leading to a non-trivial dependence of amplitudes on the initial and final states of the particles.

#### 7.1.2. High-Mass Limit

We now turn to the analysis of diagrams describing the evolution of particles in the limit m≃1. In such a regime paths are made up almost entirely of *F*-type transitions; hence, they trace a limited number of shifts. Consequently, interactions can occur when the initial sites of the particles are close to each other ($|\delta_{0}|\leq 2T-f$). The combinatorics of diagrams in this regime prevents from obtaining general formulas for $G_{int}^{\left(f_{1},f_{2}\right)}$, which can be provided only on a case-by-case basis at the lowest perturbative orders.

We begin by considering the trivial case f=2T, in which both particle paths draw straight vertical lines and thus interact at each time step (both paths must share the same initial and final sites), resulting in

$$G_{int}^{\left(T,T\right)}={\left(im\right)}^{2T}\;e^{i(T+1)\chi }\;.$$

**Figure 10:**
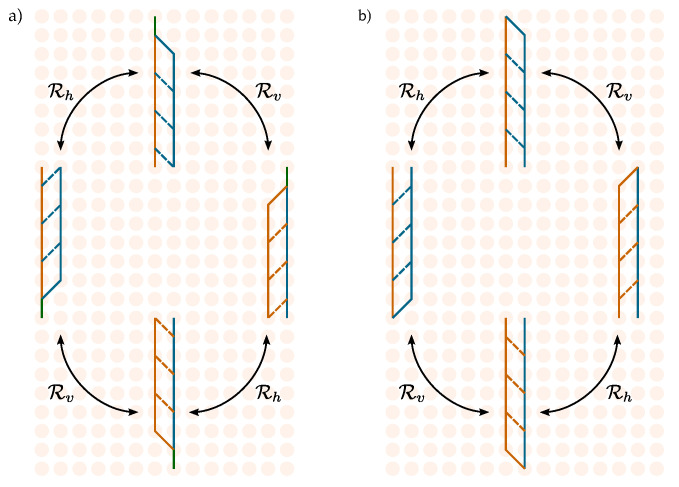
Example of diagrams belonging to the class (T,T−1) for an even number *T* of time steps. Depending on the border conditions, there can be an even (**a**) or odd (**b**) number of interactions. Green lines denote the tracks of paths both particles share, resulting in consecutive interactions. Dashed lines represent alternative paths connecting the same extremal points.

Moving to the case f=2T−1, only one particle is allowed to move once. Therefore, they must either share the same initial site and end up one site apart or vice-versa. We cannot write a closed formula for $G_{int}^{\left(T,T-1\right)}$; nonetheless, we provide an exhaustive example in Figure 10. Notice that different diagrams contributing to this perturbative order contain a different number of interactions. Their transition amplitudes are $G_{int}^{\left(T,T-1\right)}=\sum_{k=1}^{T/2}e^{i2k\chi }$ and $G_{int}^{\left(T,T-1\right)}=\sum_{k=1}^{T/2}e^{i(2k-1)\chi }$ for panels (a) and (b), respectively. Amplitudes for *T* odd can be computed analogously.

**Figure 11:**
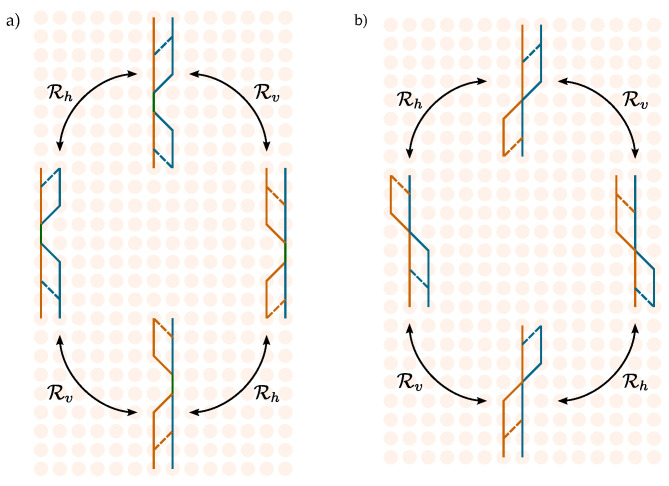
Example of diagrams belonging to the classes (**a**) (T,T−2) and (**b**) (T−1,T−1).

Lastly, the case f=2T−2 results in various classes which share no apparent common features. A couple of instances are shown in Figure 11. Their transition amplitudes are $G_{int}^{\left(T,T-2\right)}=\sum_{k=1}^{(T-2)/2}e^{i2k\chi }\;\left(T-2k\right)/2$ and $G_{int}^{\left(T-1,T-1\right)}=\sum_{k=1}^{(T-1)/2}e^{i(2k-1)\chi }\;\left(T+1-2k\right)/2$ for panels (a) and (b), respectively.

## 8. Multi-Particle Sector

This section examines the applicability of our results to the multi-particle sector, with a focus on the three-particle case. The following Lemma stems directly from Lemma 5.

**Lemma** **7.** *Within a three-particle process, if particle A can interact with particle B and particle B can interact with particle C, then particle C cannot interact with particle A.*

**Figure 12:**
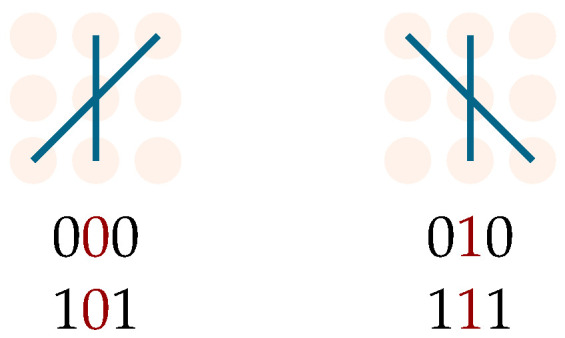
Paths violating Pauli’s exclusion principle in the multi-particle sector. Violation is highlighted in red in the two strings corresponding to the (non-interacting) paths.

This result immediately extends to an arbitrary number *N* of particles: if N−1 particles can interact with the remaining one, then they cannot interact with each other. Consequently, a significant additional complexity is introduced compared to the two-particle scenario. Specifically, for two particles with initial state within $H_{\mathrm{int}}$, any site they share on the lattice will host opposite internal degrees of freedom. On the contrary, with *N* particles, the Pauli exclusion principle can be violated as two non-interacting particles may occupy the same site with identical internal degrees of freedom (Figure 12). The corresponding diagrams are unphysical and must be excluded from amplitude calculations.

This feature of the multi-particle case renders the perturbative approach in the number of interaction vertices ineffective. Our algorithmic procedure, based on decoupling path intersections from particle interactions, inherently loses control over path intersections between interactions, making it unable to account for violations of Pauli exclusion principle.

Contrarily, the perturbative approach in the mass parameter can still be carried out. Lemma 7 proves useful within our classification procedure, especially when restricted to the three-particle sector. The classification is based on the same criteria we used for the two-particle case, with each class now identified by three integers $\left(f_{1},f_{2},f_{3}\right)$ such that $f_{1}+f_{2}+f_{3}=f$, with *f* being the chosen perturbative order. However, one must now ensure that the classification excludes unphysical diagrams containing occurrences as in Figure 12. As for the two-particle case, this procedure is feasible as long as we restrict it to the first few perturbative orders, and, unfortunately, it does not yield a general expression for the transition amplitude. A closed-form solution can be derived on a case-by-case basis. For N>3, diagram classification becomes more complex, as subsets of particles may interact among themselves but not with others.

## 9. Conclusions

The main objective of this work was to explore approximation techniques for the QCA approach to the theory of interacting quantum systems, focusing on the Thirring QCA. The latter is characterized by the most general on-site number-preserving interaction. Within the framework of the path sum formalism, adopting, in particular, a perturbative approach, we aimed to devise procedures for predicting relevant physical quantities, particularly transition amplitudes (or matrices) for a given process.

We started by building on the free theory, obtaining a few results valid for any on-site number-preserving interaction in one spatial dimension. We first showed that all binary strings corresponding to paths with given boundary conditions share the same weight. This implies that fixing the particle’s state at a given point in space–time uniquely determines its state at any other site in the space–time lattice. Upon introducing the interaction, we identified a necessary condition for initial states to comply with Pauli exclusion principle.

To address the inherent complexity of the problem, we adopted a perturbative approach in the mass parameter, which allowed us to compute the transition amplitude from some basic processes. However, our results primarily yielded a classification of all allowed diagrams in the borderline regimes of extremely light or extremely heavy particles, rather than their general characterization. Future work could extend the classification to higher perturbative orders, which might give useful insights into finding a way to reconcile the two regimes.

This work highlighted the challenges the QCA approach poses to an interacting theory, even with a simple interaction model restricted to one spatial dimension. The difficulties encountered in adopting the path sum formalism suggest it might not be the most suited framework for such an endeavor. However, this may be due to our current limited understanding of the QCA approach to interaction theory. We currently have analytical solutions only for the two-particle sector of the Thirring QCA, and its integrability for an arbitrary number of particles remains an open question. Furthermore, the peculiar structure of the discrete propagator in the path sum approach to the Dirac QCA indicates strong model dependence, meaning further investigations could lead to alternative strategies that exploit features of the model we are not yet aware of. Thus, the path sum approach may still prove valuable once future research provides us with more insights, enabling us to better handle the computational challenges we now find insurmountable.

## Data Availability

No new data were created or analyzed in this study.

## Appendix A. Dirac QCA in the Wave-Vector Representation

In wave-vector representation the Dirac QCA (3) becomes

(A1) $$W=\int_{B}dk\;\tilde{W}\left(k\right)⊗\left|k\rangle \langle k\right|\;,\;\tilde{W}\left(k\right)=\left(\begin{array}{cc} ne^{-ik} & im\\ im & ne^{ik} \end{array}\right)\;,$$

with |k〉〈k| defined on $L^{2}\left(B\right)$, where B:=[−π,π] denotes the Brillouin zone. By diagonalizing $\tilde{W}\left(k\right)$, from its eigenvalues $e^{\pm i\omega (k,m)}$, one obtains the automaton dispersion relation

$$\cos^{2}\left(\omega (k,m)\right)=\left(1-m^{2}\right)\cos^{2}\left(k\right)\;,$$

and one can write the QCA Hamiltonian $e^{-iH}=W$, which, on a wave-vector basis, reads

$$H=\int_{B}dk\;H\left(k,m\right)⊗\left|k\rangle \langle k\right|\;,\;H\left(k,m\right)=\frac{\omega }{\sin(\omega )}\left(\begin{array}{cc} n\sin(k) & m\\ m & -n\sin(k) \end{array}\right)\;.$$

Upon identifying the parameter *m* with the mass and *k* with the momentum of a particle, in the low-mass and low-momentum regime m,k→0, the evolution described by the Dirac QCA cannot be discerned from the one prescribed by the Dirac equation of quantum field theory

$$\tilde{\psi }\left(k,t\right)=U_{D}\left(t\right)\tilde{\psi }\left(k,0\right)\;,\;U_{D}\left(t\right)=e^{-iH_{D}\left(k\right)\;t}\;,\;H_{D}\left(k\right)=\left(\begin{array}{cc} k & m\\ m & -k \end{array}\right)\;,\;t\in R\;,$$

hence the name: Dirac QCA. This is achieved [34] by computing the magnitude of the error of an optimal discrimination test between the unitary operators $U_{D}$ and *W* in this regime, where as m,k→0, we have

$$H\left(k,m\right)=H_{D}\left(k\right)+O\left(k^{p}m^{q}\right)\;\;,\;\;\omega \left(k,m\right)=\sqrt{k^{2}+m^{2}}+O\left(k^{p}m^{q}\right)\;\;\mathrm{with}\;\;p+q=3\;.$$

Assuming the QCA serves as a microscopic description at high energies—hypothetically at the Planck scale ($10^{28}$ eV, with m=1 and |k|=π representing Planck mass and momentum, respectively)—the low-mass and low-momentum limit becomes physically well-justified. Any energy scale exceeding the LHC center-of-mass energy (∼$10^{13}$ eV) by two or three orders of magnitude is suitable for the large-scale limit of the Dirac QCA to align with current particle physics knowledge. Moreover, at these energies, potential deviations between QCA and QFT models might be observable through ultra-high-energy cosmic rays (recorded events reaching up to ∼$10^{20}$ eV). Indeed, the typical energy scales of current particle physics experiments are many orders of magnitude lower (∼$10^{-15}$ eV in Planck units). Thus, for sufficiently small *m*, we can fix a momentum cutoff Λ≪π such that the Dirac QCA and the standard Dirac evolution become experimentally indistinguishable. Introducing an ultraviolet momentum cutoff is akin to taking a large-scale limit of the QCA in positions representation. Importantly, in this large-scale limit, the granularity of space–time remains fixed, while the limit involves considering states with momentum below Λ. This restriction prevents the system from detecting its underlying discreteness, thereby yielding an effective continuous description. This approach is analogous to that in statistical mechanics, where the microscopic model is often a lattice model, whereas the field theory only serves as an effective description of long-distance physics. In contrast, the standard continuum limit of QFT is attained by reducing the lattice spacing and the Trotterized time step to zero, while simultaneously extending the space–time volume to infinity, to recover the continuum theory from its discretized version. This underscores how QCA-based simulations can differ from those of standard lattice QFT. We conclude by highlighting that this model avoids fermion doubling problem [34] and restores Lorentz covariance at low energies relative to the automaton scale [26].

## Appendix B. Exact Solution of the Thirring QCA in the Two-Particle Sector

We briefly recall here the solution in the two-particle sector of the Thirring QCA (see Ref. [36]). In the two-particle case, N=2, it is convenient to work in the centre-of-mass basis $|a_{1},a_{2}\rangle |y\rangle |w\rangle \in H^{⊗2}$, where $a_{i}\in \left\{R,L\right\}$, while $y=x_{1}-x_{2}$ and $w=x_{1}+x_{2}$ denote the relative position and the centre-of-mass coordinates of the particles, respectively. The interaction operator can thus be represented as

$$J\left(\chi \right)=e^{i\;\chi \;\delta_{y,0}\left(1-\delta_{a_{1},a_{2}}\right)}\;.$$

Defining k,p∈B as the (half) relative momentum $k=(k_{1}-k_{2})/2$ and the (half) total momentum $p=(k_{1}+k_{2})/2$ of the particles, respectively, the free QCA can be represented as

$$\begin{array}{c} W_{2}=\int_{B\times B}dk\;dp\;{\tilde{W}}_{2}\left(k,p\right)⊗\left|k\rangle \langle k\right|⊗\left|p\rangle \langle p\right|\;,\\ {\tilde{W}}_{2}\left(k,p\right):=\tilde{W}\left(p+k\right)⊗\tilde{W}\left(p-k\right)\;, \end{array}$$

with $\tilde{W}$ as in (A1). Since the interacting dynamics described by $U_{2}$ commutes with the translations in the center-of-mass coordinate *w*, it is convenient to write the automaton in the hybrid basis $|a_{1},a_{2}\rangle |y\rangle |p\rangle$

$$\begin{array}{c} U_{2}=\int_{B}dp\;U_{2}\left(\chi ,p\right)⊗\left|p\rangle \langle p\right|\;,\;U_{2}:=\left(\chi ,p\right)W_{2}^{h}\left(p\right)J^{h}\left(\chi \right)\\ W_{2}^{h}\left(p\right):=mn\left(\begin{array}{cccc} \frac{n}{m}e^{-i2p} & ie^{-ip}T_{y} & ie^{-ip}T_{y}^{†} & -\frac{m}{n}\\ ie^{-ip}T_{y} & \frac{n}{m}T_{y}^{2} & -\frac{m}{n} & ie^{ip}T_{y}\\ ie^{-ip}T_{y}^{†} & -\frac{m}{n} & {\frac{n}{m}T_{y}^{†}}^{2} & ie^{ip}T_{y}^{†}\\ -\frac{m}{n} & ie^{ip}T_{y} & ie^{ip}T_{y}^{†} & \frac{n}{m}e^{i2p} \end{array}\right)\;J^{h}\left(\chi \right)\left(\begin{array}{cccc} I & 0 & 0 & 0\\ 0 & e^{i\;\chi \;\delta_{y,0}}I & 0 & 0\\ 0 & 0 & e^{i\;\chi \;\delta_{y,0}}I & 0\\ 0 & 0 & 0 & I \end{array}\right) \end{array}$$

where the superscript *h* stands for “hybrid”, and $T_{y}$ and $\;T_{y}^{†}$ are the right and left shift in the relative coordinate *y*. Solving the two-particle dynamics amounts to the diagonalization of the infinite-dimensional operator $U_{2}\left(\chi ,p\right)$. In Ref. [36], it is shown that the eigenvalue problem

$$U_{2}\left(\chi ,p\right)\psi =e^{i\omega }\psi$$

can be suitably reduced to a finite set of algebraic equations that admit an analytical solution. This is achieved by considering the linear difference equation

$$\begin{array}{cc} U_{2}\left(\chi ,p\right)\; & f_{p,\omega ,\chi }\left(y\right)=e^{i\omega }\;f_{p,\omega ,\chi }\left(y\right)\\ \; & f_{p,\omega ,\chi }\;:\;Z\to C^{4}\;\;\;,\;\;\;\omega \in C \end{array}$$

for any possible value of χ and *p*. Since, in the new coordinates, the interacting term acts only at the origin, for y>0, such an equation results in a linear recurrence relation with constant coefficients, which are then determined by requiring the solutions to be anti-symmetric eigenvectors (or generalized eigenvectors) of $U_{2}\left(\chi ,p\right)$ intended as an operator on the Hilbert space $C^{4}⊗l^{2}\left(Z\right)$. The solutions thus obtained present some rather peculiar features:

- There exist bound states for every value of the total momentum and of the coupling constant; even in the case of vanishing coupling, the non-interacting walk manifests bound states for finitely many isolated values of the total momentum;
- In addition to the two conventional scattering solutions of quantum field theory, i.e., elastic bounce and quantum tunneling, there are two other classes of scattering solutions with nontrivial momentum transfer involving jumps between distant regions of the Brillouin zone.

The large-scale limit discussed for the Dirac QCA (Appendix A), which successfully recovers the Dirac equation, is not suitable for the treatment of interactions; thus, we do not expect that it yields the Thirring model if applied to the Thirring QCA. Specifically, the large-scale limit can be thought of as a step of some renormalization group. Renormalization theory describes how the description of degrees of freedom and interactions of a model change across different physical scales. In the case of the Dirac QCA, the renormalized large-scale degrees of freedom are represented by low-momentum states, with large-scale dynamics captured by the low-mass, low-momentum limit of *H*. Unlike free models, where the relevant physical scale is set by mass and momentum observables alone, in interacting models, the coupling strength becomes an additional scale-defining parameter, requiring a large-scale limit handling these interactions. Different prescriptions to implement this limit or to provide an effective continuous description can be devised.

## Appendix C. Proofs of Results

### Appendix C.1. Proof of Lemma 1

**Proof.** Let $b,b^{\prime }\in {\left\{0,1\right\}}^{3}$ be two binary strings and assume that they are mapped to the same string of translations, namely, $M_{b_{1}b_{2}}M_{b_{2}b_{3}}=M_{b_{1}^{\prime }b_{2}^{\prime }}M_{b_{2}^{\prime }b_{3}^{\prime }}$. This amounts to the following:

$$\left\{\begin{array}{c} M_{b_{1}b_{2}}=M_{b_{1}^{\prime }b_{2}^{\prime }}\\ M_{b_{2}b_{3}}=M_{b_{2}^{\prime }b_{3}^{\prime }}\;, \end{array}\right.$$

which, in turn, implies

$$\left\{\begin{array}{c} b_{1}=b_{1}^{\prime },\;b_{2}=b_{2}^{\prime }\;\vee \;b_{1}=\bar{b_{1}^{\prime }},\;b_{2}=\bar{b_{2}^{\prime }}\\ b_{2}=b_{2}^{\prime },\;b_{3}=b_{3}^{\prime }\;\vee \;b_{2}=\bar{b_{2}^{\prime }},\;b_{3}=\bar{b_{3}^{\prime }}\;, \end{array}\right.$$

depending on whether they correspond to a L,R-type transition or an *F* one. The only admissible solution is $b=b^{\prime }$. □

### Appendix C.2. Proof of Lemma 2

**Proof.** Since any permutation of bits Π can be regarded as the composition of swaps between first-neighbour bits, it is sufficient to prove the statement for the case Π=φ, with the swap map φ

$$\begin{array}{cc} \varphi \;\;:\;\;{\left\{0,1\right\}}^{2} & \to {\left\{0,1\right\}}^{2}\\ b_{1}b_{2} & \mapsto \varphi \left(b_{1}b_{2}\right)=b_{2}b_{1}\;. \end{array}$$

One can check that

(A2) $$M\circ \varphi =M,\;\;\;\;\;\mathrm{namely},\;\;\;\;\;M_{b_{1}b_{2}}=M_{b_{2}b_{1}}\;.$$

The exchange of two bits can influence, at most, three elements in the corresponding string of translations, which amounts to a length-four binary sub-string, whose two central bits are the ones affected by the swap map. Thus, given $b=b_{1}b_{2}b_{3}b_{4}\in B_{\bar{x}}^{T}$, it is sufficient to prove that $b_{1}\varphi \left(b_{2}b_{3}\right)b_{4}=b^{\prime }\;\in B_{\bar{x}}^{T}$. The scenario under scrutiny can be represented as follows:

In the formula, we want to show that

$$\Delta \left(M_{b_{1}b_{2}}\right)+\Delta \left(M_{b_{2}b_{3}}\right)+\Delta \left(M_{b_{3}b_{4}}\right)=\Delta \left(M_{b_{1}b_{3}}\right)+\Delta \left(M_{b_{3}b_{2}}\right)+\Delta \left(M_{b_{2}b_{4}}\right)\;,$$

that is, by using Equation (A2) and the definition of Δ (Equation (10)),

(A3) $$\Delta \left(M_{b_{1}b_{2}}\right)+\Delta \left(M_{b_{3}b_{4}}\right)=\Delta \left(M_{b_{1}b_{3}}\right)+\Delta \left(M_{b_{2}b_{4}}\right)\;.$$

For $b_{2}=b_{3}$, Equation (A3) is trivially satisfied. The only relevant case is then $b_{2}={\bar{b}}_{3}=b$, and Equation (A3) becomes

(A4) $$\Delta \left(M_{b_{1}b}\right)+\Delta \left(M_{\bar{b}b_{4}}\right)=\Delta \left(M_{b_{1}\bar{b}}\right)+\Delta \left(M_{bb_{4}}\right)\;.$$

One then has the following possible scenarios:

- $b_{1}=b_{4}=a$: Equation (A4) becomes
  $$\Delta \left(M_{ab}\right)+\Delta \left(M_{\bar{b}a}\right)=\Delta \left(M_{a\bar{b}}\right)+\Delta \left(M_{ba}\right),$$
  and it is satisfied, since $M_{ab}=M_{ba}$;
- $b_{1}={\bar{b}}_{4}=a$: Equation (A4) becomes
  $$\Delta \left(M_{ab}\right)+\Delta \left(M_{\bar{b}\bar{a}}\right)=\Delta \left(M_{a\bar{b}}\right)+\Delta \left(M_{b\bar{a}}\right),$$

and it is satisfied, since $\Delta \left(M_{\bar{a}\bar{b}}\right)=-\Delta \left(M_{ab}\right)$. □

### Appendix C.3. Proof of Lemma 3

**Proof.** Consider the string of transitions $s=s_{1}\ldots s_{T}$ associated with a path connecting the state $|b_{0}^{x},\;x_{0}\rangle ,|b_{T}^{x},\;x_{T}\rangle \in H$ in *T* time steps. Let f,r, and *l* denote the number of F,R, and *L* type transitions contained within such a string. To compute the weight—namely, the number of ones—of the corresponding binary string $b_{0}^{x}\ldots b_{T}^{x}=b\in B_{\bar{x}}^{T}$ associated with the internal degrees of freedom, we need to consider the following:

- Each *F* transition contributes one, each *L* transition contributes two, while *R* transitions do not contribute at all (see Definition 1).
- Each transition $s_{i}$ identifies a pair of bits $b_{i-1}b_{i}$ in the binary string *b*. The bit $b_{i-1}$ is shared with the previous transition $s_{i-1}$ and the second bit $b_{i}$ is shared with the following one $s_{i+1}$. Therefore, to avoid over-counting, the contribution of each transition to the weight must be divided by 2.
- The previous observation does not hold for the first and last elements of the string *s*. Thus, we add the contribution of the first and last bits by hand before dividing.

It follows that the weight of the binary string *b* is given by

(A5) $$w\left(b\right)=\frac{2l+f+b_{0}^{x}+b_{T}^{x}}{2}\;.$$

By exploiting the following equations (Ref. [35]):

$$x_{0}-x_{T}=l-r\;\;\;\;\;\;T=r+l+f,$$

Equation (A5) becomes

$$w\left(b\right)=\frac{T+x_{0}-x_{T}+b_{0}^{x}+b_{T}^{x}}{2}\;.$$

□

### Appendix C.4. Proof of Lemma 4

**Proof.** From the weight formula (Equation (11)), by fixing $x_{0}$ and $b_{0}^{x}$ while treating $x_{T}$ and $b_{T}^{x}$ as variables, since the numerator must be even, one can write

$$b_{T}^{x}=\left[T+x_{0}-x_{T}+b_{0}^{x}\right]\;\mathrm{mod}2\;\;\;\;\;\forall \;x_{T}\;,$$

thus proving the statement. □

### Appendix C.5. Proof of Lemma 5

**Proof.** Let us denote two particles as A and B. Let $x_{0}$ and $y_{0}=x_{0}+\delta_{0}$ be the space coordinates of the two particles at time t=0, and let (v,t) be the space–time coordinates of the site where they interact. The weights of the binary strings $b^{A}=b_{0}^{A}\cdots b_{t}^{A}$ and $b^{B}=b_{0}^{B}\cdots \bar{b_{t}^{A}}$, corresponding to paths connecting $x_{0}$ and $y_{0}$ to *v*, respectively, are given by (Equation (11))

$$\begin{array}{cc} w(b^{A}) & =\frac{t+x_{0}-v+b_{0}^{A}+b_{t}^{A}}{2}\;,\\ w(b^{B}) & =\frac{t+y_{0}-v+b_{0}^{B}+\bar{b_{t}^{A}}}{2}\;, \end{array}$$

where the internal degrees of freedom of the particles are opposite when both are at the interaction site (according to Pauli exclusion principle and Thirring interaction). It follows that

$$\begin{array}{cc} b_{0}^{A} & =\left[t+x_{0}-v+b_{t}^{A}\right]\;\mathrm{mod}2\\ b_{0}^{B} & =\left[t+y_{0}-v+\bar{b_{t}^{A}}\;\right]\;\mathrm{mod}2\\ \; & =\left[t+\left(x_{0}+\delta_{0}\right)-v+\left(1+b_{t}^{A}\right)\right]\;\mathrm{mod}2\\ \; & =\delta_{0}⊕\left[1+\left(t+x_{0}-v+b_{t}^{A}\right)\right]\;\mathrm{mod}2\\ \; & =\delta_{0}⊕\bar{b_{0}^{A}}\;. \end{array}$$

This leads to

$$\left\{\begin{array}{cc} b_{0}^{B}=\bar{b_{0}^{A}}\;\;\;\;\; & if\;\;\;\;\;\delta_{0}\;\;even\\ b_{0}^{B}=b_{0}^{A}\;\;\;\;\; & if\;\;\;\;\;\delta_{0}\;\;odd\;. \end{array}\right.$$

Lastly, in Ref. [52], it is shown that the projector $P_{int}$ onto the interacting subspace $H_{int}\subset H^{⊗2}$, which can be represented in the hybrid basis as

$$P_{int}=\left(\begin{array}{cccc} P_{o} & 0 & 0 & 0\\ 0 & P_{e} & 0 & 0\\ 0 & 0 & P_{e} & 0\\ 0 & 0 & 0 & P_{o} \end{array}\right)\;\mathrm{with}\;P_{e}:=\sum_{y\in Z}|2y\rangle \langle 2y|\;,\;P_{o}:=\sum_{y\in Z}|2y+1\rangle \langle 2y+1|\;,$$

commutes with the dynamics $U_{2}$, which thus preserves the interacting subspace. □

### Appendix C.6. Proof of Corollary 1

**Proof.** We prove the statement by contradiction. Let $b^{x}\in B_{\bar{x}}^{T}$ and $b^{y}\in B_{\bar{y}}^{T}$ with $|b_{0}^{x},\;x_{0}\rangle |b_{0}^{y},\;y_{0}\rangle \in H_{\mathrm{int}}$. If a string *a* connecting $|b_{0}^{x},\;x_{0}\rangle$ to $|b_{T}^{y},\;y_{T}\rangle$ existed, then $w\left(a\right)\notin N_{0}$, similarly to a string connecting $|b_{0}^{y},\;y_{0}\rangle$ to $|b_{T}^{x},\;x_{T}\rangle$. □

### Appendix C.7. Proof of Lemma 6

**Proof.** Let *A* and *B* denote two particles and $b^{A},\;b^{B}$ their paths. Let $\delta_{t}=x_{t}^{A}-x_{t}^{B}$ be the relative position of particles at time *t* and assume without loss of generality that $x_{t-1}^{A}\leq x_{t-1}^{B}$. Let us evaluate the relative displacement of each particle associated with the transition leading up to an interaction at the site (v,t). Since at the interaction site the particles must have opposite internal degrees of freedom, one has

$$\begin{array}{cc} \Delta \left(M_{b_{t-1}^{A}\;0}\right) & =1-b_{t-1}^{A}\;,\\ \Delta \left(M_{b_{t-1}^{B}\;1}\right) & ={\left(-1\right)}^{b_{t-1}^{B}}\;b_{t-1}^{B}\;. \end{array}$$

It follows that

$$\Delta \left(M_{b_{t-1}^{A}\;0}\right)\geq \Delta \left(M_{b_{t-1}^{B}\;1}\right)\;\;\;\;\;\forall \;b_{t-1}^{A},b_{t-1}^{B}\in \left\{0,1\right\}\;.$$

As $M_{ab}=M_{ba}$, for the transition after the interaction, we have

$$\Delta \left(M_{0\;b_{t+1}^{A}}\right)\geq \Delta \left(M_{1\;b_{t+1}^{B}}\right)\;\;\;\;\;\forall \;b_{t+1}^{A},b_{t+1}^{B}\in \left\{0,1\right\}\;.$$

Since

$$\begin{array}{cc} \Delta \left(M_{b_{t-1}^{B}\;1}\right)-\Delta \left(M_{b_{t-1}^{A}\;0}\right) & =\delta_{t-1}\;,\\ \Delta \left(M_{0\;b_{t+1}^{A}}\right)-\Delta \left(M_{1\;b_{t+1}^{B}}\right) & =\delta_{t+1}\;, \end{array}$$

we obtain $\delta_{t-1}\delta_{t+1}\leq 0$. □

### Appendix C.8. Proof of Corollary 2

**Proof.** Let us define the function θ:R→{−1,1}

$$\theta \left(x\right)=\left\{\begin{array}{cc} 1\; & \mathrm{if}\;x>0\\ -1\; & \mathrm{if}\;x\leq 0\;. \end{array}\right.$$

It follows from Lemma 6 that if an interaction occurs at step *t*, then

$$\theta \left(\delta_{t-2}\delta_{t}\right)\;\theta \left(\delta_{t-1}\delta_{t+1}\right)\;\theta \left(\delta_{t}\delta_{t+2}\right)=-1\;.$$

Thus, upon denoting by *n* the number of interactions, it is immediate to check that

$$\theta \left(\delta_{0}\delta_{T}\right)=\prod_{i=1}^{T-1}\theta \left(\delta_{i-1}\delta_{i+1}\right)={\left(-1\right)}^{n}\;.$$

□

### Appendix C.9. Proof of Lemma 7

**Proof.** Let us denote with $x_{0}^{A},x_{0}^{B},x_{0}^{C}$ and $b_{0}^{A},b_{0}^{B},b_{0}^{C}$ the initial spatial coordinate and internal degree of freedom of particles A,B,C, respectively. By hypothesis, let particle *B* interact with both particle *A* and particle *C*. From Lemma 5, it follows that

$$\left\{\begin{array}{cc} b_{0}^{B}=\bar{b_{0}^{i}}\;\;\;\;\; & \mathrm{if}\;\;\;\;\;\delta_{0}^{i}\;\;\mathrm{even}\\ b_{0}^{B}=b_{0}^{i}\;\;\;\;\; & \mathrm{if}\;\;\;\;\;\delta_{0}^{i}\;\;\mathrm{odd} \end{array}\right.\;\;\;\;\;i=A,C\;,$$

where $\delta_{0}^{i}=x_{B}-x_{i}$, i=A,C. The relative position between particles *A* and *C* can thus be evaluated as $\Omega =\delta_{0}^{C}-\delta_{0}^{A}$ (the sign is irrelevant since we are only interested in its parity). All possible scenarios are summarized in Table A1.

**Table A1:** Relations between the relative positions of particles *A* and *C* with respect to B and their internal degrees of freedom.

	$\delta_{0}^{C}$ Even	$\delta_{0}^{C}$ Odd
$\delta_{0}^{A}$ even	Ω even ∧ $\bar{b_{0}^{A}}=\bar{b_{0}^{C}}$	Ω odd ∧ $\bar{b_{0}^{A}}=b_{0}^{C}$
$\delta_{0}^{A}$ odd	Ω odd ∧ $b_{0}^{A}=\bar{b_{0}^{C}}$	Ω even ∧ $b_{0}^{A}=b_{0}^{C}$ No entry of the table satisfies the necessary condition (Lemma 5) for particles *A* and *C* to be able to interact. □
